# Genetically predicted inflammatory proteins and the risk of atrial fibrillation: a bidirectional Mendelian randomization study

**DOI:** 10.3389/fcvm.2024.1375750

**Published:** 2024-06-26

**Authors:** Zhiqiang Ma, Qiao Chen, Ziyuan Liu, Xueyu Li, Huaming Zhang, Xi Feng

**Affiliations:** Division of Cardiology, Departments of Internal Medicine, Liyuan Hospital, Tongji Medical College, Huazhong University of Science and Technology, Wuhan, China

**Keywords:** inflammatory proteins, atrial fibrillation, Mendelian randomization, genetic, finnGen

## Abstract

**Purpose:**

The causal associations between inflammatory factors and atrial fibrillation (AF) remained unclear. We aimed to investigate whether genetically predicted inflammatory proteins are related to the risk of AF, and vice versa.

**Methods:**

A bidirectional two-sample Mendelian randomization study was performed. The genetic variation of 91 inflammatory proteins were derived from genome-wide association study (GWAS) data of European ancestry (*n* = 14,824). Summary statistics for AF were obtained from a published meta-analysis study (*n* = 1,030,836) and the FinnGen study (*n* = 261,395).

**Results:**

Genetically predicted fibroblast growth factor 5 (FGF5) was significantly positively associated with risk of AF [[odds ratio (OR): 1.07; 95% CI: 1.04–1.10; *P *< 0.01], and CD40l receptor was significantly negatively associated with risk of AF (OR: 0.95; 95% CI: 0.92–0.98; *P* = 0.02) in the meta-analysis study. In the FinnGen study, similar results were observed in FGF5 (OR: 1.11; 95% CI: 1.06–1.16; *P *< 0.01) and CD40l receptor (OR: 0.93; 95% CI: 0.89–0.97; *P* = 0.03) for AF. In the FinnGen study, TNF-beta was significantly positively associated with risk of AF (OR: 1.05; 95% CI: 1.02–1.09; *P* = 0.03) and leukemia inhibitory factor receptor was significantly negatively associated with risk of AF (OR: 0.86; 95% CI: 0.80–0.91; *P* = 0.001). The causal effect of AF on inflammatory proteins was not observed.

**Conclusion:**

Our study suggested that FGF5 and CD40l receptor have a potential causal association with AF, and targeting these factors may help in the treatment of AF.

## Introduction

Atrial fibrillation (AF) is the most common clinical arrhythmia, with an incidence of 2%–5% (1, 2). It is estimated that 15.9 million people will have AF in the United States by 2050 and 17.9 million in Europe by 2060 (3, 4). Despite optimal contemporary therapy with anticoagulation and rate control strategies, patients with AF were associated with adverse cardiac and cerebrovascular events, such as stroke, heart failure, myocardial infarction, and sudden death (2). Therefore, novel therapeutic modalities are needed to improve the prognosis of patients with AF.

In addition to the traditional risk factors such as hypertension, diabetes, smoking, and obesity, inflammatory factors play a crucial role in AF (5). Several studies have assessed the specific contributions to AF development of inflammatory signaling pathways in animal models. In animals with sepsis, increased atrial infiltration of inflammatory macrophages and CD68^+^ cells were observed, which contribute to the vulnerability of AF (6). The underlying mechanisms are complex and likely related to the reduced L-type Ca2^+^ current and increased potassium current. In addition, atrial cardiomyocytes could also produce potent pro-inflammatory cytokines, such as interleukin (IL)-1β, IL-6, IL-18, and tumor necrosis factor (TNF), further amplifying the inflammatory signal and its propagation (7, 8). This suggested that circulating inflammatory cytokines could potentially be one of the mechanisms underlying the AF development. A similar phenomenon was also observed in patients with AF. In specimens of atrial tissue from patients with AF, compared to patients with sinus rhythm, increased inflammatory cells, such as CD68-KP1^+^ inflammatory cells, CD45^+^ cells and CD3^+^ T-lymphocytes, have been confirmed (9, 10). In addition, numerous observational studies have found significant associations between AF development and disease with systemic or local inflammation, such as sepsis, rheumatoid arthritis, psoriasis, Crohn's disease, or pericarditis (11–15).

Whether inflammation is a cause or consequence of AF is still uncertain. Causality is difficult to establish based on observational studies due to residual confounding from unknown or unmeasured factors and reverse causality. Mendelian randomization (MR) is an epidemiologic technique that utilizes genetic variants that are reliably associated with a potentially modifiable risk factor to determine its causal role for disease risk (16, 17). Using genetic variants as instrumental variables for an exposure, the MR design can strengthen the causal inference by minimizing residual confounding and reverse causation.

Understanding the pathogenesis of genetic variants underlying the increased AF risk in inflammatory factors can ultimately provide insight into the immune and inflammatory components of AF, as well as revealing opportunities for targeted therapeutics. The aim of this study was to explore association between AF and 91 inflammatory protein levels by MR analysis.

## Methods

### Study design and overview

**Figure 1:**
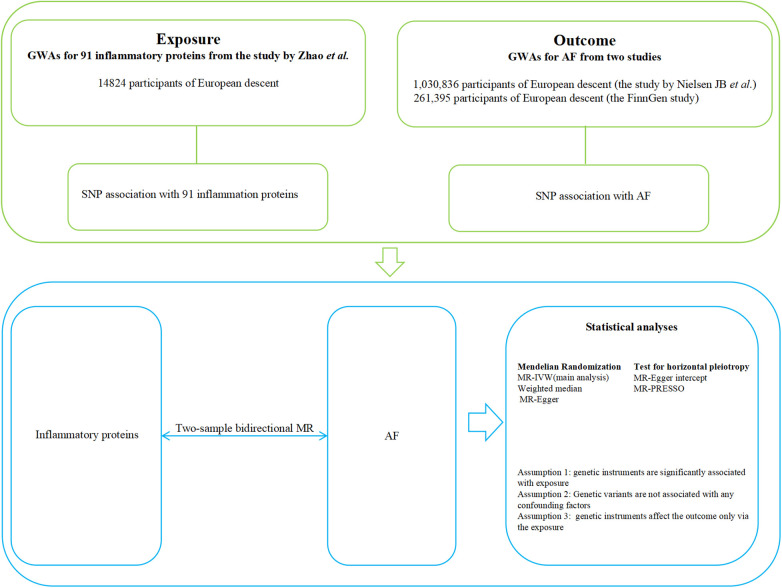
Overview of the study design in this bidirectional MR study. AF, atrial fibrillation; GWAS, genome-wide association studies; SNP, single nucleotide polymorphisms.

This bidirectional two-sample MR study adheres to the Reporting of Observational Studies in Epidemiology Using Mendelian Randomization STROBE Guidelines (18). A schematic overview of the bidirectional two-sample MR study design is detailed in Figure 1. Briefly, the design of the study was as follows: (1) genome-wide association study (GWAS) data for 91 inflammatory proteins and AF were retrieved from independent samples avoiding bias due to overlapping; (2) suitable instrumental variables, namely single nucleotide polymorphisms (SNPs), were derived from the corresponding GWAS mentioned above and satisfied the correlation, independence, and exclusivity assumptions; (3) defining 91 inflammatory proteins as exposure and AF as the outcome, a two-sample MR analysis was conducted to assess causal effect; (4) considering the possibility of reverse causality, we further evaluated the causal effect of AF on 91 inflammatory proteins; (5) after obtaining results, a two-sample MR analysis was conducted using another GWAS dataset of AF to ensure the stability of the prediction results.

### MR assumptions

MR depends on three key assumptions: (1) genetic instruments are significantly associated with exposure of interest; (2) genetic instruments are not related to any confounding factors of the exposure-outcome association; (3) genetic instruments affect the outcome only via the exposure (19).

### Data sources

Supplementary Material Table S1 summarizes the details of GWAS included in our study. The data regarding the 91 circulating inflammatory proteins were obtained from the reanalyzed outcomes of a serum cytokine-associated GWAS, which can be accessed at https://www.phpc.cam.ac.uk/ceu/proteins/ (20). This GWAS study consists of 11 cohorts, totaling of 14,824 individuals of European ancestry. For AF, GWAS of 60,620 cases and 970,216 controls of Eeuropean ancestry were included in our study (21). In this study, the average age of patients at initial diagnosis of AF is 65–76 years old, with 53% being female, approximately 75% have hypertension, approximately 19% have diabetes, approximately 31% have had myocardial infarction, and approximately 37% have heart failure (Supplementary Material Table S2). The AF patients were all diagnosed according to the International Classification of Disease (ICD) codes, ICD-9 or ICD-10. For the replication analysis, summary-level data for AF were collected from the FinnGen study, consisting of 261,395 individuals (50,743 cases and 210,652 controls) (22). The mean age of subjects at initial AF diagnosis is 68.11 years, with 37% of the total 18,932 individuals being female (Supplementary Material Table S2). The FinnGen study is a large-scale genomics initiative that has analyzed over 500,000 Finnish biobank samples and correlated genetic variation with health data to understand disease mechanisms and predispositions (https://www.finngen.fi/en/for_researchers). All the GWAS included in the present study obtained written informed consent from participants and were approved by ethics committees. No further ethical consents were required since our study is based on publicly available summary-level data.

### Genetic instruments selection

The steps for selecting optimal genetic instruments were as follows: (1) at the beginning, we establish a threshold of *P* < 5 × 10^−8^ as the significant level across the entire genome in order to identify SNPs that are highly correlated with 91 inflammatory proteins. Due to the limited number of SNPs detected in relation to cytokines as the exposure, a relatively relaxed threshold (*P* < 5 × 10^−6^) was chosen employed in MR analysis. In the reverse direction, independent instruments of AF (*P* < 5 × 10^−8^) identified from the original GWAS were used as instruments; (2) the linkage disequilibrium of instrumental variables was removed to ensure mutual independence of these instrumental variables (*r*^2^ = 0.001, kb = 10,000); (3) to quantify the strength of instrumental variables, we calculated *F*-statistics, and a threshold of the *F*-statistics >10 was typically recommended for MR analyses; (4) We examined several potential confounders, such as obesity, hypertension, and coronary artery disease, all chosen on the basis of those reported in previous literature (5). Then, the PhenoScanner database (http://www.phenoscanner.medschl.cam.ac.uk/about/) was employed for the purpose of searching and screening SNPs associated with confounding factors. These SNPs with confounding factors were subsequently excluded from MR analysis to ensure the reliability and consistency of the results.

### Statistical analyses

The primary analysis for the MR study was the inverse-variance weighted (IVW) method, which provides a robust causal estimate in the absence of directional pleiotropy. Supplementary analyses were conducted using the weighted median and MR-Egger methods (23). The weighted median method can provide consistent estimates when more than 50% of the weight comes from valid instrument variants (24). MR-Egger regression can generate estimates after accounting for horizontal pleiotropy albeit with less precision (25). If the IVW method result is significant (*P* < 0.05), even if the results of other methods are not significant, and no pleiotropy and heterogeneity was identified, it can be regarded as a positive result, provided that the beta values of the other methods are in the same direction. To correct for multiple comparisons for multiple hypotheses, a false discovery rate (FDR) adjusted *p*-value was used in the main IVW MR analyses (*P* < 0.05 was judged significant) (26). Then, we performed tests for directional horizontal pleiotropy by MR-Egger intercept and MR-PRESSO (*P *< 0.05 and was judged significant). As pleiotropic effects of genetic variants will lead to overdispersion in the MR-Egger regression model, heterogeneity between the causal estimates is expected, and so a random-effects analysis should always be preferred when using MR-Egger (27). If heterogeneity is absent, then a random-effects analysis is equivalent to a fixed-effect analysis. To assess the heterogeneity for MR-Egger regression and IVW method, Cochran's Q Test was employed and random-effects models will be used for all analyses. The study utilized R 4.2.2 software and the R packages “TwosampleMR” and “MR-PRESSO” for analysis.

## Results

### Genetic instruments

When utilizing the 91 inflammatory proteins for exposure, a selection threshold of *P* < 5 × 10^−6^ was adopted. During the screening process with confounding factors, 50 instrumental variables were excluded (Supplementary Material Table S3). Overall, 2–30 instruments were included in the final analysis. Among instrumental variables included in the final analysis, all *F*-values are greater than 10, indicating that weak instrument bias is unlikely to be significant. When utilizing AF for exposure, 80–99 instruments were included in the final analysis and all *F*-values are greater than 10.

### Estimates of causal effect of inflammatory proteins on AF

**Figure 2:**
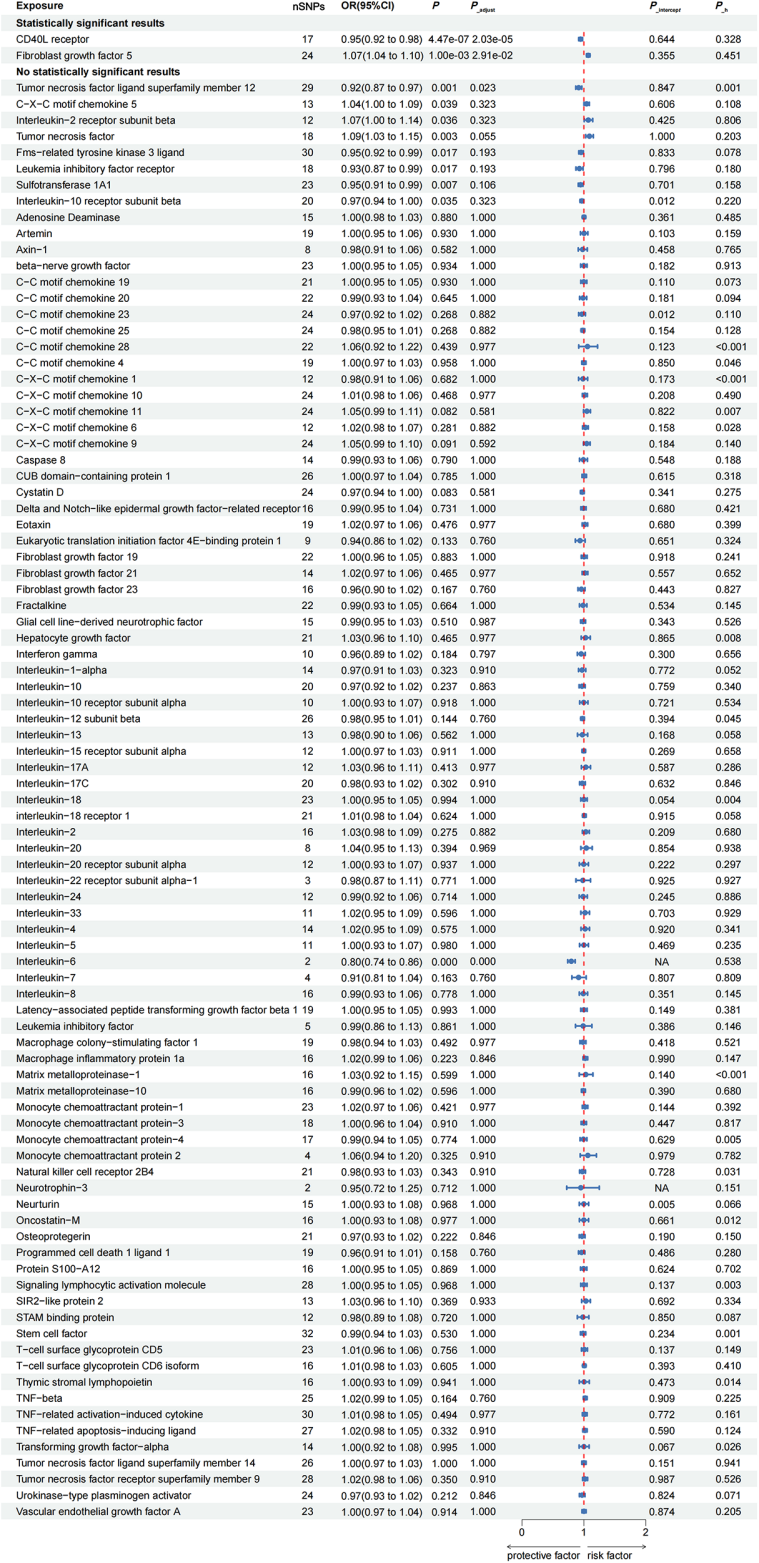
Causal correlations of 91 inflammatory proteins on atrial fibrillation. Genetically predicted higher levels of circulating CD40l receptor and FGF5 were associated with the risk of AF, when GWAS of AF from the study by Nielsen JB et al*.* were used as outcome. AF, atrial fibrillation; CI, confidence interval; NA, insufficient SNPs for MR analysis; OR, odds ratio; SNP, single nucleotide polymorphism.

In the GWAS of AF from the study by Nielsen JB et al., after FDR adjustment, two inflammatory proteins were identified as causal cytokines associated with AF based on IVW method (Figure 2). Genetically predicted higher levels of circulating fibroblast growth factor 5 (FGF5) were associated with an increased risk of AF (OR: 1.07; 95% CI: 1.04–1.10; *P*__adjust_ < 0.001). The direction of the β-values of IVW, MR-Egger and weighted median were consistent (Table 1).

**Table 1:** Genetic predicted inflammatory proteins on the risk of AF in the MR analysis.

Exposure	Data source of AF	Methods	β	*P*	OR (95%CI)	MR-PRESSO	*P*__intercept_	*P*__h_
FGF-5 levels	Nielsen JB et al. (21)	MR Egger	0.08	4.66e-04	1.08 (1.04–1.13)	0.92	0.35	0.44
		Inverse variance weighted	0.08	2.91e-02*	1.07 (1.04–1.10)			0.45
		Weighted median	0.06	1.77e-07	1.09 (1.05–1.12)			
	Kurki MI et al. (22)	MR Egger	0.13	1.14e-03	1.14 (1.06–1.22)	0.06	0.30	0.01
		Inverse variance weighted	0.10	4.69e-04*	1.11 (1.06–1.16)			0.01
		Weighted median	0.14	6.60e-11	1.15 (1.10–1.20)			
CD40l receptor levels	Nielsen JB et al. (21)	MR Egger	−0.04	7.09e-02	0.95 (0.91–1.00)	0.53	0.64	0.28
		Inverse variance weighted	−0.05	2.03e-05*	0.95 (0.92–0.98)			0.32
		Weighted median	−0.05	2.86e-03	0.95 (0.91–0.98)			
	Kurki MI et al. (22)	MR Egger	−0.06	4.43e-02	0.93 (0.88–0.99)			0.97
		Inverse variance weighted	−0.07	3.17e-02*	0.93 (0.89–0.97)	0.99	0.92	0.98
		Weighted median	−0.06	5.29e-03	0.93 (0.89–0.98)			

* *P*-values were adjusted by false discovery rate; AF, atrial fibrillation; OR, odds ratio; CI, confidence interval.

In addition, there was no evidence of pleiotropy (MR-Egger *P*__intercept_ = 0.355; MR-PRESSO *P*__mr−presso_ = 0.391) and heterogeneity (*P*__heterogeneity_ = 0.451) (Table 1). Among all SNPs in FGF5, the most relevant SNP with AF was rs1902859 (β = 8.25E-02; *P* = 3.42E-07; OR: 1.09; 95%CI: 1.05–1.12) (Supplementary Material Table S4). In addition, IVW results showed that genetically predicted increases CD40l receptor levels were negatively associated with AF risk (OR: 0.95; 95% CI: 0.92–0.98; *P*__adjust_ = 0.029), and no evidence of pleiotropy or heterogeneity was observed (*P__intercept_* = 0.644; *P*__mr−presso_ = 0.539; *P*__heterogeneity_ = 0.328). Among all SNPs in CD40l receptor, the most relevant SNP with AF was rs12624433 (β = −5.47E-02; *P* = 2.94E-03; OR: 0.95; 95%CI: 0.91–0.98) (Supplementary Material Table S4). The scatter plots and funnel plots of MR analyses are exhibited in Supplementary Material Figure S1,S2. The funnel plot showed slight asymmetry, which means potential discrepancy in SNP distributions. The leave-one-out analysis demonstrated the stability of the results.

**Figure 3:**
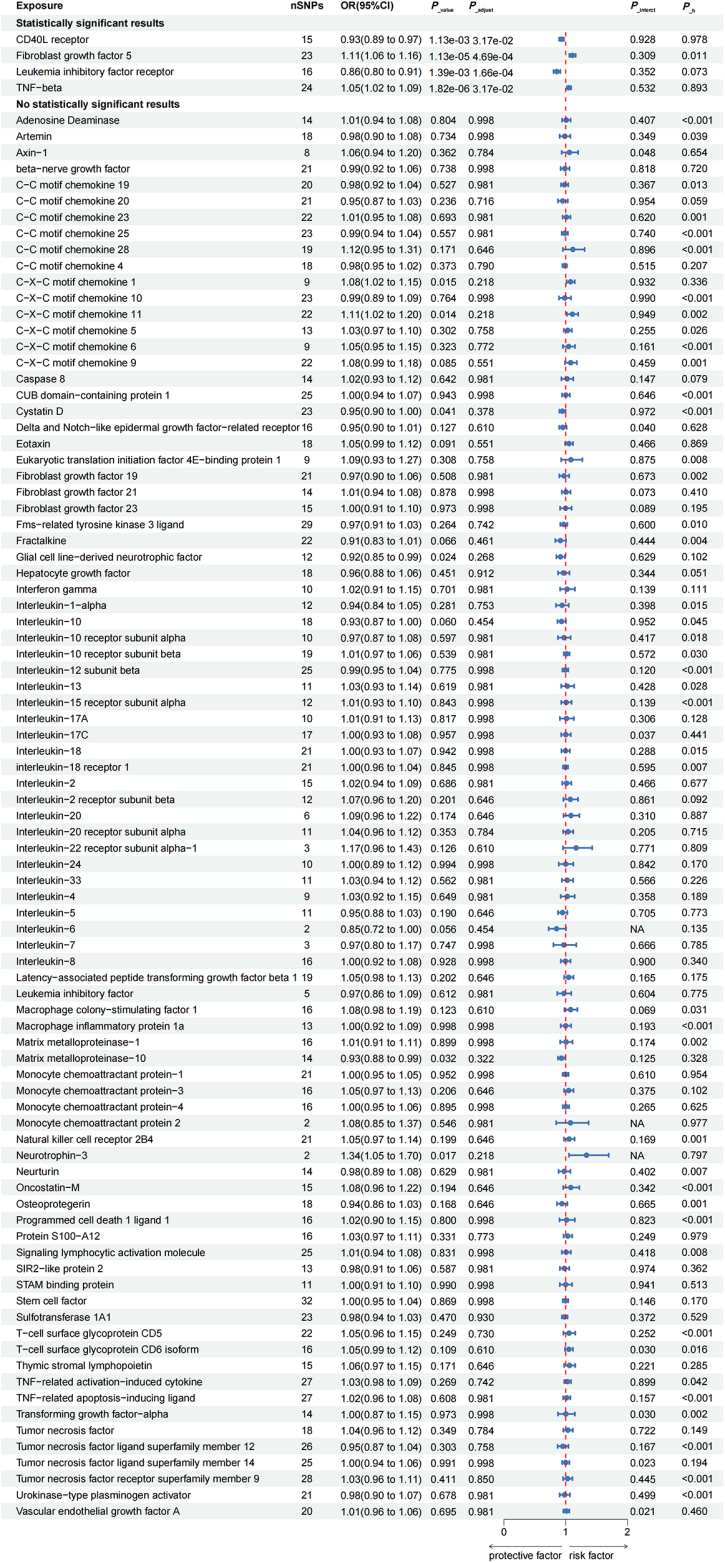
Secondary validation of the causal correlations of 91 inflammatory proteins on atrial fibrillation. Genetically predicted higher levels of circulating CD40l receptor, FGF5, leukemia inhibitory factor receptor and TNF-beta were associated with the risk of AF, when GWAS of AF from the FinnGen study were used as outcome. AF, atrial fibrillation; CI, confidence interval; NA, insufficient SNPs for MR analysis; OR, odds ratio; SNP, single nucleotide polymorphism.

To ensure the stability of the prediction results, the GWAS of AF from the FinnGen study was used as outcome, and a two-sample MR analysis was performed as shown in Figure 3. In the GWAS from the FinnGen study, IVW results showed that increases FGF5 levels were associated with an increased risk of AF and were no evidence of pleiotropy (OR: 1.11; 95% CI: 1.07–1.16; *P* < 0.001; *P*__intercept_ = 0.15; *P*__mr−presso_ = 0.20). The direction of the β-values of IVW, MR-Egger and weighted median were consistent (Table 1). However, there was significant heterogeneity *(P*__heterogeneity_ = 0.02). In single SNP analysis, the most relevant SNP with AF was also rs1902859 (β = 1.50E-01; *P* = 5.61E-12; OR: 1.16; 95%CI: 1.11–1.21) (Supplementary Material Table S4). The scatter plots and funnel plots of MR analyses are exhibited in Supplementary Material Figure S3. The funnel plots analysis showed symmetry. The leave-one-out analysis demonstrated the stability of the results. The same causal relationship was observed between TNF-β levels and AF risk (OR: 1.05; 95% CI: 1.02–1.09; *P*__adjust_ = 0.03). In addition, a significantly negative association with the risk of AF was observed in CD40l receptor levels (OR: 0.93; 95% CI: 0.89–0.97; *P*__adjust_ = 0.03; *P*__intercept_ = 0.74; *P*__mr−presso_ = 0.96; *P*__heterogeneity_ = 0.97) and leukemia inhibitory factor receptor levels (OR: 0.86; 95% CI: 0.80–0.91; *P*__adjust_ < 0.001). Among all SNPs in CD40l receptor, the most relevant SNP with AF was also rs12624433 (β = −6.88E-02; *P* = 7.39E-03; OR: 0.93; 95%CI: 0.89–0.98) (Supplementary Material Table S4). The direction of the β-values of IVW, MR-Egger and weighted median were consistent (Table 1). The scatter plots and funnel plots of MR analyses for CD40l receptor levels in AF are exhibited in Supplementary Material Figure S4. The funnel plots analysis showed symmetry. The leave-one-out analysis demonstrated the stability of the results. The all results of the main MR analyses for the 91 cytokines are presented in Supplementary Material Table S5,S6.

### Estimates of causal effect of AF on inflammatory proteins

When AF were used as exposures, the main results of the MR analysis are shown in Supplementary Material Figure S5,S6. In all analyses, there were no observed causal relationships between AF and inflammation proteins. The all results of the main MR analyses are presented in Supplementary Material Table S7,S8.

## Discussion

In this bidirectional two-sample MR study, after a series of stringent quality control measures, we identified two inflammation proteins (fibroblast growth factor 5 levels and CD40l receptor levels) that may suggestively be the upstream causes of the AF development. In turn, when AF is considered as the exposure variable, there were no observed causal relationships between inflammation proteins and AF. Our study has provided genetic insight between inflammation proteins and AF and may reveal novel targets for AF therapy and prevention.

CD40l receptor, a member of the tumor necrosis factor receptor superfamily, acts as a receptor upon activation by its classical ligands (CD40 ligand, CD40l). It is widely expressed on B cells, T cells, platelets, monocytes, macrophages, and smooth muscle cells (28–30). The CD40-CD40l system is the hub of immune response and inflammatory response, and the serum levels of both increase simultaneously in pathological conditions (31). Despite its role as an inflammatory mediator, soluble CD40l (sCD40l) is mainly derived from activated platelets and triggers clot formation. In previous studies, elevated preoperative levels of sCD40l reflected overall platelet activation, and were associated with a higher risk of developing AF after off-pump CABG surgery (32, 33). In addition, numerous observational studies have found elevated levels of sCD40l in AF patients, and could predict thrombus formation as well as stroke in AF patients prospectively (34–36). This appears to indicate that levels of sCD40l were a risk factor for patients with AF. However, the majority of patients in these studies have coronary artery disease, or have underlying diseases, including hypertension, coronary heart disease, or diabetes. It was shown that the above-mentioned chronic diseases could also elevate levels of sCD40l, which may lead to bias (37). In addition, other potential confounders were present, such as arrhythmia episode or AF duration, the effects of medications on sCD40l levels and laboratory test errors (38). In this ground, the casual correlations of AF with the CD40-CD40l system are still unclear due to the limitations of classical epidemiology. In our study, the increased CD40l receptor levels were associated with a decrease in AF risk, which suggested that the increased CD40l receptor levels may be a protective response for patients with AF. Although this result is inconsistent with the findings of previously observed studies, MR analysis is a more feasible strategy compared to observational studies in the presence of many confounders. For example, in recently MR studies, as opposed to previously observed studies, the increased CD40l receptor levels were associated with a lower risk of large artery stroke (39, 40). Thus, the function of CD40l receptor in AF is worthy of further study.

FGF5, a member of the fibroblast growth factor superfamily, is involved in multiple human biological processes, such as cell growth, morphogenesis, tumor growth and invasion, tissue repair, and inflammatory processes (41). Earlier studies have reported the potential protective cardiovascular effects of FGF5 (42–44). However, these effects were only applicable in a porcine model of stress-induced myocardial ischemia. In addition, it was shown that circulating FGF5 levels were associated with chronic inflammatory diseases, such as hypertension, spinal cord injury, malignances, and hepatic fibrosis (45–48). Currently, there are only a few studies related to FGF5 in AF. In our study, even after excluding SNPs associated with confounding factors from MR analysis, higher levels of circulating FGF5 were associated with an increased risk of AF. This finding could suggest that FGF5 has a causative and potentially prognostic role in patients with AF. Thus, targeting FGF5 may be beneficial for the treatment of AF. More clinical and basic studies are required to further determine the relationship between them.

In the past decades, the role of inflammation in the pathophysiology of AF has been suspected, and considerable evidence has subsequently accrued. However, the existence of inflammatory signalling in cardiomyocytes and its pathophysiological importance in AF have been recognized only for the past 5 years. A series of studies have evaluated the association between inflammatory factors and atrial structural changes, and their involvement in the occurrence and development of AF (49–51). In our study, FGF5 was associated with an increased risk of AF. One previous study suggested that FGF5 elicited prominent effects on myocyte cell death, cell growth, and hypertrophy in animal models. The authors showed that this effect may be related to the mobilization of progenitor cells or endogenous cardiac stem cells by FGF5, as well as the disruption of the balance between cell death and cell growth/regeneration. Myocardial hypertrophy may lead to the pathophysiology of AF through abnormal calcium handling, causing ectopic triggers from delayed afterdepolarisations. Therefore, we speculate cautiously that the mechanism by which FGF5 leads to AF may be related to its promotion of myocardial hypertrophy. As a key player in immunity, previous studies have shown that the CD40-CD40l interaction was primarily investigated in connection with T-cell activation, B-cell proliferation and differentiation and switching of antibodies from IgM to IgG (29, 52). Immune disorders may lead to changes in cardiac structure, resulting in electrophysiological disturbances (53). Although the mechanism remains unclear, elevated levels of sCD40l have been found to be associated with atrial structural changes in observational studies among patients with AF (35, 36). Further studies are warranted to illuminate the mechanistic landscapes of FGF5 and CD40l receptor in AF, and expedite research on inflammation-related AF treatments.

In our study, TNF-beta and leukemia inhibitory factor receptor were statistically significant only in the FinnGen study but were not validated in the study by Nielsen JB et al. There could be several reasons for this discrepancy. Firstly, the different population characteristics included in the two GWAS studies have reduced the consistency of the results to some extent. To maximize statistical power, the GWAS data for the study by Nielsen JB et al*.* were obtained from 7 different study cohorts, with statistical results primarily driven by deCODE and UKB cohorts. In comparison to the FinnGen study, the proportion of female patients is higher in the two mentioned cohorts (49% and 54% vs. 37%), and the total sample size is larger than that of the FinnGen study. Secondly, the FinnGen study does not only include patients with AF, but also includes some patients with atrial flutter. Although there is an association between AF and atrial flutter, differences in their pathogenesis and pathophysiology still exist. This bias may also affect the results, leading to discrepancies. Thirdly, differences in the original cohort study designs and inclusion/exclusion criteria can also lead to the inconsistent findings. Larger studies are needed to further clarify the roles of TNF-beta and leukemia inhibitory factor receptor in AF.

The bilateral MR analysis in this study showed that AF may not be correlated with changes in 91 inflammation proteins. Until now, studies on the effect of AF on inflammation factors were inconclusive. In animal models, rapid atrial-pacing was reported to cause the elevation of inflammation cytokines such as IL-6, TNF or NLR family pyrin domain containing 3 inflammasome (7, 54). In addition, previous studies showed that C-reactive protein and IL-6 serum levels were significantly decreased in patients with atrial flutter after successful ablation (55). A study by Yamazoe et al*.* suggested that mitochondrial-cfDNA, a biomarker of inflammation, may be involved in sterile systemic inflammation accompanied by AF (56). However, as previously mentioned, every observational study was limited by the potential for confounding factors. In addition, MR analysis was limited by the availability of GWAS. Based on our results, it is currently difficult to prove whether AF is the cause of inflammation. One possible answer is that inflammation has been identified as a significant catalyst for the onset of AF, while AF seems to foster an environment conducive to inflammation.

Immunomodulatory therapy for AF has attracted attention in recent years and become a new therapeutic trend. The NOD-like receptor family pyrin-domain containing-3 (NLRP3) inflammasome has recently attracted the attention of researchers due to its unique pro-inflammatory effect in AF. Activation of the NLRP3 inflammasome promotes the secretion of IL-1β and IL-18, which further aggravates inflammation (57). In previous clinical trials, colchicine, a non-selective NLRP3 inhibitor, has been investigated to prevent the recurrence of AF after catheter ablation for AF and to reduce the risk of AF after cardiac surgery (58, 59). This suggests that colchicine has a strong potential as an anti-inflammatory drug to be used in AF patients. Currently, daily dosage of 0.6 mg colchicine is also investigated as a therapeutic agent to reduce the risk of AF after ablation in a phase III clinical trial (NCT05459974) (60). Additionally, some studies have explored the possibility of targeting interleukins as therapeutic targets for AF. For example, targeting IL-6, IL-10 and transforming growth factor-β, affects the occurrence and development of atrial fibrillation in animal models (51–62). In a recent randomized controlled study, 24 patients with AF were randomly assigned to receive a single subcutaneous injection of 150 mg of canakinumab (a fully human monoclonal antibody targeting the IL-1β) or matching placebo after electrical cardioversion. The results showed that AF recurrence at 6 months occurred in 10 (77%) and 4 (36%) patients in the placebo and canakinumab groups, respectively (*P *= 0.09). Although the results were not statistically significantly different, they emphasize the potential of anti-inflammatory treatments to reduce the recurrence rate of AF (63).

Safety must always be a primary consideration when assessing new therapeutic strategies targeting inflammation cytokines. In previous studies, some anti-inflammatory drugs, such as canakinumab (a fully human monoclonal antibody targeting the IL-1β) and methotrexate (a systemic anti- inflammatory drug targeting TNF-α), were limited in clinical practice by frequent infection events (64, 65). Hence, implementing anti-inflammatory treatment for AF in regular clinical care requires safe and effective medication. Sotigalimab, a CD40 agonist monoclonal antibody, showed good safety in patients with pancreatic adenocarcinoma (66, 67). In the phase II clinical trial, the most common non-hematologic treatment-related adverse events of any grade were nausea, fatigue, pyrexia and chills. In addition, only 2 (6%) patients receiving sotigalimab treatment discontinued treatment owing to adverse events (pneumonitis and pyrexia), and 2 patients (6%) died due to an adverse event (acute hepatic failure and intracranial hemorrhage). There are few studies on the safety of FGF5. In a recent study by Amano et al. showed that some RNA aptamers have high affinity and specificity for FGF5 and inhibit FGF5-induced cell proliferation (68). However, there is no study reporting on clinical results.

Our study has some limitations. (1) It is important to note that the FinnGen data included some patients with atrial flutter. Despite the close relationship between AF and atrial flutter, there are still fundamental differences between the two in terms of pathophysiology and pathogenesis. This inconsistency among the study population could increase the potential for bias, thereby impacting the generalizability and comparability of the research findings. Although we used data from two studies for validation analysis to minimize this bias as much as possible, this remains one of the biggest limitations of our study; (2) although we explored the association between CD40l receptor, FGF5, and AF from a genetic perspective, the underlying mechanisms are not clear, and further prospective randomized large-scale studies and basic studies are required to further determine our results; (3) although we excluded SNPs related to obesity, hypertension, and CAD, some other risk factors could not be completely eliminated, which might limit the stability of our results; (4) the two-sample MR methods rely on GWAS summary statistics and assume a linear relationship between the exposure and the outcome. We did not evaluate a potential nonlinear relationship between 91 inflammation proteins and AF; (5) in addition, the duration of AF in all GWAS studies was not analyzed; currently, there is an unclear association between the duration of AF and the levels of inflammation proteins; (6) the GWAS data used in the study were all from European populations, indicating that the results of this study may not be applicable to individuals of other ancestries. (7) additionally, the majority of patients included in the study are elderly with an average age of 65–76 years. Therefore, the conclusions of this study should be interpreted with caution as they may not be generalizable to a wider population. Due to the differences in the characteristics of the population included in different cohorts, this study may not apply to other characteristic populations.

In conclusion, our bidirectional MR study indicated a causal link between FGF5 or CD40l receptor and AF, and the reverse direction showed no causal associations. Thus, targeting FGF5 or CD40l receptor may be beneficial for treating AF.

## Data Availability

The original contributions presented in the study are included in the article/**Supplementary Material**, further inquiries can be directed to the corresponding authors.
