# Supplementary material for: Genetically predicted inflammatory proteins and the risk of atrial fibrillation: a bidirectional Mendelian randomization study
Source: Front Cardiovasc Med. 2024 Jun 26;11:1375750. doi: 10.3389/fcvm.2024.1375750 (PMC11234858; doi:10.3389/fcvm.2024.1375750)
Supplement: Supplementary file 1 [file Datasheet1.docx]

**Supplemental materials**

**Content**

**Supplementary tables**

Table S1. Details of the data sources used in this study

Table S2. SNPs excluded due to confounding factors

Table S3. MR estimates of 91 inflammatory proteins on AF (Summary statistics of AF were obtained from the study by Nielsen JB *et al*.)

Table S4. MR estimates of 91 inflammatory proteins on AF (Summary statistics of AF were obtained from the FinnGen study)

Table S5. MR estimates of AF on inflammatory proteins (Summary statistics of AF were obtained from the study by Nielsen JB *et al*.)

Table S6. MR estimates of AF on inflammatory proteins (Summary statistics of AF were obtained from the FinnGen study)

**Supplementary figures**

**Figure S1.** Mendelian randomization (MR) analysis of fibroblast growth factor 5 and AF, when GWAS of AF from the study by Nielsen JB *et al.* were used as outcome. (A) Scatter plot of MR analysis; (B) Forest plot of MR analysis; (C) Funnel plots of MR analysis; (D) leave-one-out sensitivity analysis of MR analysis.

**Figure S2.** Mendelian randomization (MR) analysis of fibroblast growth factor 5 and AF, when GWAS of AF from the FinnGen study were used as outcome. (A) Scatter plot of MR analysis; (B) Forest plot of MR analysis; (C) Funnel plots of MR analysis; (D) leave-one-out sensitivity analysis of MR analysis.

**Figure S3.** Mendelian randomization (MR) analysis of CD40L receptor and AF, when GWAS of AF from the study by Nielsen JB *et al.* were used as outcome. (A) Scatter plot of MR analysis; (B) Forest plot of MR analysis; (C) Funnel plots of MR analysis; (D) leave-one-out sensitivity analysis of MR analysis.

**Figure S4.** Mendelian randomization (MR) analysis of CD40L receptor and AF, when GWAS of AF from the FinnGen study were used as outcome. (A) Scatter plot of MR analysis; (B) Forest plot of MR analysis; (C) Funnel plots of MR analysis; (D) leave-one-out sensitivity analysis of MR analysis.

**Figure S5.** Causal correlations of atrial fibrillation on 91 inflammatory proteins, when GWAS of AF from the study by Nielsen JB *et al.* were used as exposure. AF, atrial fibrillation; CI, confidence interval; OR, odds ratio; SNP, single nucleotide polymorphism.

**Figure S6.** Secondary validation of the causal correlations of atrial fibrillation on 91 inflammatory proteins, when GWAS of AF from the FinnGen study were used as exposure. AF, atrial fibrillation; CI, confidence interval; OR, odds ratio; SNP, single nucleotide polymorphism.

**Supplementary Methods**

Pleiotropy is the phenomenon by which the same gene can result in multiple phenotypes and there are two types of pleiotropy: horizontal and vertical pleiotropy. The validity of Mendelian randomization results mainly depends on pleiotropy, specifically “horizontal pleiotropy” whereby a genetic variant affects the outcome through a pathway that does not involve the risk factor of interest ^1^. The presence of horizontal pleiotropy violates the third assumption of Mendelian randomization, leading to a spurious, non-causal association between exposure and outcome. In our study, we conducted a pleiotropy analysis using two statistical methods including the MR-Egger intercept test and the MR pleiotropy residual sum and outlier (MR-PRESSO) test (*P* < 0.05 is considered to indicate significant pleiotropy).

Heterogeneity in a Mendelian randomization study refers to the variation between instrumental variable. In this study, we used Cochran’s Q test to quantify heterogeneity among the instrument variables ((*P* < 0.05 is considered to indicate heterogeneity). According to previous study, if heterogeneity exists, a random effects model should be used; If it does not, using a random effects model is equivalent to a fixed effects model ^2^.

1. Larsson SC, Butterworth AS, Burgess S. Mendelian randomization for cardiovascular diseases: principles and applications. Eur Heart J. 2023;44(47):4913-4924. doi:10.1093/eurheartj/ehad736
2. Yuan S, Kim JH, Xu P, Wang Z. Causal association between celiac disease and inflammatory bowel disease: A two-sample bidirectional Mendelian randomization study. Front Immunol. 2023;13:1057253. Published 2023 Jan 4. doi:10.3389/fimmu.2022.1057253

| **Table S1. Details of the data sources used in this study** | | | |
| --- | --- | --- | --- |
| Factors | PMID | Samples | Cohorts (number of patients included the study) |
| Inflammatory cytokines | 37563310 | 14824 | The INTERVAL study (4896)  The STABILITY study (2951)  The BioFINDER study (1496)  The KORA F4 study (1064)  The ORCADES study (981)  The NSPHS study (866)  The CROATIA-Vis study (899)  The SWEBIC study (644)  The EstBB study (487)  The EIRA study (540)  The ARISTOTLE study (1585) |
| Atrial fibrillation | 30061737 | 1030836 | The Nord-Trøndelag Health Study (69635)  deCODE (371632)  The Michigan Genomics Initiative (12275)  DiscovEHR (48482)  UK Biobank (395739)  The AFGen Consortium (133073) |
|  | 36653562 | 261,395 | The FinnGen study (261,395) |
|  | | | |

| **Table S2. SNPs excluded due to confounding factors** | | | |
| --- | --- | --- | --- |
| SNP | Effect Allele | Other Allele | Trait |
| rs3733402 | A | G | obesity |
| rs2731672 | T | C | coronary artery disease |
| rs12075 | A | G | obesity |
| rs4766578 | A | T | coronary artery disease and hypertension |
| rs4907572 | A | G | coronary artery disease |
| rs635634 | T | C | coronary artery disease and hypertension |
| rs521795 | C | T | hypertension |
| rs679574 | C | G | hypertension |
| rs2631360 | G | A | hypertension |
| rs3184504 | C | T | coronary artery disease and hypertension |
| rs597808 | G | A | coronary artery disease and hypertension |
| rs12290068 | A | G | coronary artery disease |
| rs516316 | G | C | hypertension |
| rs2442752 | T | C | coronary artery disease |
| rs6937696 | C | T | hypertension |
| rs12770839 | A | C | hypertension |
| rs13107325 | T | C | obesityand hypertension |
| rs112126444 | A | G | coronary artery disease |
| rs516246 | T | C | obesityand hypertension |
| rs4970834 | C | T | coronary artery disease |
| rs1902859 | C | T | coronary artery disease and hypertension |
| rs59950280 | A | G | coronary artery disease |
| rs9266257 | C | G | coronary artery disease and hypertension |
| rs11039216 | C | T | hypertension |
| rs649129 | T | C | coronary artery disease and hypertension |
| rs7137828 | C | T | coronary artery disease and hypertension |
| rs28929474 | T | C | coronary artery disease |
| rs11066309 | A | G | coronary artery disease and hypertension |
| rs141460846 | C | T | hypertension |
| rs12900168 | A | G | coronary artery disease |
| rs2228145 | A | C | coronary artery disease |
| rs2631367 | G | C | hypertension |
| rs7013321 | A | G | coronary artery disease |
| rs2584662 | C | A | coronary artery disease and hypertension |
| rs6985207 | A | C | hypertension |
| rs112635299 | G | T | coronary artery disease |
| rs653178 | T | C | coronary artery disease and hypertension |
| rs7125631 | C | T | hypertension |
| rs2980888 | T | C | coronary artery disease |
| rs10832027 | A | G | hypertension |
| rs12149545 | A | G | coronary artery disease |
| rs7310615 | C | G | coronary artery disease and hypertension |
| rs11950562 | A | C | hypertension |
| rs4760 | G | A | coronary artery disease |
| rs117113213 | A | G | coronary artery disease |
| rs579459 | C | T | coronary artery disease and hypertension |
| rs34790908 | C | T | hypertension |
| rs154972 | G | A | coronary artery disease |
| rs6993770 | A | T | coronary artery disease |
| rs10822155 | A | C | hypertension |

| **Table S3. MR estimates of 91 inflammatory proteins on AF** | | | | | | | |
| --- | --- | --- | --- | --- | --- | --- | --- |
| **Exposure** | **SNPs** | **Inverse variance weighted** | | **MR Egger** | | **Weighted median** | |
|  |  | *P* | OR (95%CI) | *P* | OR(95%CI) | *P* | OR(95%CI) |
| CD40L receptor | 17 | <0.001 | 0.95(0.92 to 0.98) | 0.071 | 0.95(0.91 to 1.00) | 0.003 | 0.95(0.91 to 0.98) |
| Fibroblast growth factor 5 | 24 | 0.001 | 1.07(1.04 to 1.10) | <0.001 | 1.08(1.04 to 1.13) | 0.001 | 1.09(1.05 to 1.12) |
| Tumor necrosis factor ligand superfamily member 12 | 29 | 0.001 | 0.92(0.87 to 0.97) | 0.212 | 0.93(0.82 to 1.04) | 0.060 | 0.93(0.87 to 1.00) |
| C-X-C motif chemokine 5 | 13 | 0.039 | 1.04(1.00 to 1.09) | 0.322 | 1.03(0.97 to 1.10) | 0.042 | 1.04(1.00 to 1.08) |
| Fms-related tyrosine kinase 3 ligand | 30 | 0.017 | 0.95(0.92 to 0.99) | 0.239 | 0.96(0.90 to 1.03) | 0.308 | 0.98(0.93 to 1.02) |
| Interleukin-2 receptor subunit beta | 12 | 0.036 | 1.07(1.00 to 1.14) | 0.829 | 1.02(0.88 to 1.17) | 0.149 | 1.06(0.98 to 1.15) |
| Leukemia inhibitory factor receptor | 18 | 0.017 | 0.93(0.87 to 0.99) | 0.453 | 0.94(0.82 to 1.09) | 0.402 | 0.97(0.90 to 1.04) |
| Sulfotransferase 1A1 | 23 | 0.007 | 0.95(0.91 to 0.99) | 0.157 | 0.93(0.84 to 1.02) | 0.016 | 0.93(0.87 to 0.99) |
| Tumor necrosis factor | 18 | 0.003 | 1.09(1.03 to 1.15) | 0.169 | 1.09(0.97 to 1.22) | 0.051 | 1.07(1.00 to 1.15) |
| Adenosine Deaminase | 15 | 0.880 | 1.00(0.98 to 1.03) | 0.697 | 0.99(0.96 to 1.03) | 0.436 | 0.99(0.96 to 1.02) |
| Artemin | 19 | 0.930 | 1.00(0.95 to 1.06) | 0.138 | 0.89(0.77 to 1.03) | 0.906 | 1.00(0.93 to 1.08) |
| Axin-1 | 8 | 0.582 | 0.98(0.91 to 1.06) | 0.378 | 0.92(0.77 to 1.10) | 0.330 | 0.96(0.87 to 1.05) |
| beta-nerve growth factor | 23 | 0.934 | 1.00(0.95 to 1.05) | 0.235 | 1.08(0.95 to 1.23) | 0.785 | 0.99(0.92 to 1.06) |
| C-C motif chemokine 19 | 21 | 0.930 | 1.00(0.95 to 1.05) | 0.177 | 0.95(0.87 to 1.02) | 0.459 | 0.98(0.93 to 1.03) |
| C-C motif chemokine 20 | 22 | 0.645 | 0.99(0.93 to 1.04) | 0.160 | 0.90(0.78 to 1.04) | 0.443 | 0.97(0.90 to 1.05) |
| C-C motif chemokine 23 | 24 | 0.268 | 0.97(0.92 to 1.02) | 0.083 | 1.10(0.99 to 1.23) | 0.877 | 0.99(0.93 to 1.07) |
| C-C motif chemokine 25 | 24 | 0.268 | 0.98(0.95 to 1.01) | 0.815 | 1.00(0.96 to 1.05) | 0.236 | 0.98(0.95 to 1.01) |
| C-C motif chemokine 28 | 22 | 0.439 | 1.06(0.92 to 1.22) | 0.308 | 0.85(0.64 to 1.15) | 0.577 | 0.98(0.90 to 1.06) |
| C-C motif chemokine 4 | 19 | 0.958 | 1.00(0.97 to 1.03) | 0.864 | 1.00(0.95 to 1.05) | 0.527 | 0.99(0.96 to 1.02) |
| C-X-C motif chemokine 1 | 12 | 0.682 | 0.98(0.91 to 1.06) | 0.193 | 0.93(0.83 to 1.03) | 0.165 | 0.97(0.92 to 1.01) |
| C-X-C motif chemokine 10 | 24 | 0.468 | 1.01(0.98 to 1.06) | 0.529 | 0.98(0.91 to 1.05) | 0.323 | 1.03(0.97 to 1.09) |
| C-X-C motif chemokine 11 | 24 | 0.082 | 1.05(0.99 to 1.11) | 0.615 | 1.03(0.91 to 1.18) | 0.018 | 1.07(1.01 to 1.14) |
| C-X-C motif chemokine 6 | 12 | 0.281 | 1.02(0.98 to 1.07) | 0.086 | 1.06(1.00 to 1.13) | 0.058 | 1.03(1.00 to 1.07) |
| C-X-C motif chemokine 9 | 24 | 0.091 | 1.05(0.99 to 1.10) | 0.608 | 0.97(0.86 to 1.09) | 0.192 | 1.04(0.98 to 1.11) |
| Caspase 8 | 14 | 0.790 | 0.99(0.93 to 1.06) | 0.516 | 0.95(0.83 to 1.10) | 0.201 | 0.95(0.88 to 1.03) |
| CUB domain-containing protein 1 | 26 | 0.785 | 1.00(0.97 to 1.04) | 0.570 | 1.02(0.95 to 1.09) | 0.375 | 1.02(0.97 to 1.07) |
| Cystatin D | 24 | 0.083 | 0.97(0.94 to 1.00) | 0.689 | 0.99(0.95 to 1.04) | 0.125 | 0.97(0.94 to 1.01) |
| Delta and Notch-like epidermal growth factor-related receptor | 16 | 0.731 | 0.99(0.95 to 1.04) | 0.607 | 0.97(0.89 to 1.07) | 0.258 | 0.97(0.91 to 1.02) |
| Eotaxin | 19 | 0.476 | 1.02(0.97 to 1.06) | 0.487 | 1.03(0.94 to 1.13) | 0.302 | 1.03(0.97 to 1.10) |
| Eukaryotic translation initiation factor 4E-binding protein 1 | 9 | 0.133 | 0.94(0.86 to 1.02) | 0.854 | 0.98(0.80 to 1.20) | 0.282 | 0.94(0.85 to 1.05) |
| Fibroblast growth factor 19 | 22 | 0.883 | 1.00(0.96 to 1.05) | 0.969 | 1.00(0.88 to 1.13) | 0.635 | 0.98(0.92 to 1.05) |
| Fibroblast growth factor 21 | 14 | 0.465 | 1.02(0.97 to 1.06) | 0.820 | 0.99(0.89 to 1.10) | 0.826 | 1.01(0.95 to 1.07) |
| Fibroblast growth factor 23 | 16 | 0.167 | 0.96(0.90 to 1.02) | 0.766 | 1.03(0.86 to 1.24) | 0.799 | 0.99(0.91 to 1.07) |
| Fractalkine | 22 | 0.664 | 0.99(0.93 to 1.05) | 0.463 | 0.94(0.80 to 1.10) | 0.849 | 0.99(0.92 to 1.07) |
| Glial cell line-derived neurotrophic factor | 15 | 0.510 | 0.99(0.95 to 1.03) | 0.687 | 1.01(0.95 to 1.09) | 0.719 | 0.99(0.94 to 1.04) |
| Hepatocyte growth factor | 21 | 0.465 | 1.03(0.96 to 1.10) | 0.860 | 1.01(0.87 to 1.19) | 0.267 | 0.96(0.88 to 1.03) |
| Interferon gamma | 10 | 0.184 | 0.96(0.89 to 1.02) | 0.869 | 1.01(0.90 to 1.14) | 0.364 | 0.96(0.88 to 1.05) |
| Interleukin-1-alpha | 14 | 0.323 | 0.97(0.91 to 1.03) | 0.772 | 0.98(0.88 to 1.10) | 0.512 | 0.98(0.91 to 1.05) |
| Interleukin-10 | 20 | 0.237 | 0.97(0.92 to 1.02) | 0.822 | 0.99(0.88 to 1.11) | 0.778 | 0.99(0.92 to 1.06) |
| Interleukin-10 receptor subunit alpha | 10 | 0.918 | 1.00(0.93 to 1.07) | 0.801 | 1.02(0.90 to 1.16) | 0.930 | 1.00(0.91 to 1.09) |
| Interleukin-10 receptor subunit beta | 20 | 0.035 | 0.97(0.94 to 1.00) | 0.747 | 1.01(0.97 to 1.05) | 0.393 | 0.99(0.96 to 1.02) |
| Interleukin-12 subunit beta | 26 | 0.144 | 0.98(0.95 to 1.01) | 0.826 | 0.99(0.95 to 1.04) | 0.774 | 0.99(0.96 to 1.03) |
| Interleukin-13 | 13 | 0.562 | 0.98(0.90 to 1.06) | 0.139 | 0.84(0.69 to 1.04) | 0.356 | 0.96(0.87 to 1.05) |
| Interleukin-15 receptor subunit alpha | 12 | 0.911 | 1.00(0.97 to 1.03) | 0.396 | 1.02(0.97 to 1.07) | 0.717 | 1.01(0.97 to 1.04) |
| Interleukin-17A | 12 | 0.413 | 1.03(0.96 to 1.11) | 0.842 | 0.98(0.81 to 1.19) | 0.311 | 1.05(0.95 to 1.16) |
| Interleukin-17C | 20 | 0.302 | 0.98(0.93 to 1.02) | 0.420 | 0.95(0.83 to 1.08) | 0.352 | 0.97(0.91 to 1.04) |
| Interleukin-18 | 23 | 0.994 | 1.00(0.95 to 1.05) | 0.086 | 1.10(0.99 to 1.22) | 0.453 | 1.02(0.96 to 1.08) |
| interleukin-18 receptor 1 | 21 | 0.624 | 1.01(0.98 to 1.04) | 0.695 | 1.01(0.97 to 1.05) | 0.462 | 1.01(0.98 to 1.04) |
| Interleukin-2 | 16 | 0.275 | 1.03(0.98 to 1.09) | 0.432 | 0.94(0.82 to 1.09) | 0.486 | 1.03(0.95 to 1.10) |
| Interleukin-20 | 8 | 0.394 | 1.04(0.95 to 1.13) | 0.545 | 1.05(0.90 to 1.23) | 0.920 | 1.01(0.90 to 1.12) |
| Interleukin-20 receptor subunit alpha | 12 | 0.937 | 1.00(0.93 to 1.07) | 0.261 | 0.91(0.78 to 1.06) | 0.216 | 0.94(0.86 to 1.04) |
| Interleukin-22 receptor subunit alpha-1 | 3 | 0.771 | 0.98(0.87 to 1.11) | 0.876 | 0.96(0.63 to 1.46) | 0.893 | 0.99(0.86 to 1.14) |
| Interleukin-24 | 12 | 0.714 | 0.99(0.92 to 1.06) | 0.238 | 0.92(0.80 to 1.05) | 0.402 | 0.96(0.89 to 1.05) |
| Interleukin-33 | 11 | 0.596 | 1.02(0.95 to 1.09) | 0.596 | 1.06(0.87 to 1.29) | 0.884 | 0.99(0.91 to 1.08) |
| Interleukin-4 | 14 | 0.575 | 1.02(0.95 to 1.09) | 0.762 | 1.03(0.86 to 1.22) | 0.774 | 0.99(0.90 to 1.08) |
| Interleukin-5 | 11 | 0.980 | 1.00(0.93 to 1.07) | 0.520 | 1.06(0.90 to 1.25) | 0.671 | 1.02(0.94 to 1.11) |
| Interleukin-6 | 2 | <0.000 | 0.80(0.74 to 0.86) | NA | NA | NA | NA |
| Interleukin-7 | 4 | 0.163 | 0.91(0.81 to 1.04) | 0.934 | 0.98(0.60 to 1.59) | 0.151 | 0.90(0.77 to 1.04) |
| Interleukin-8 | 16 | 0.778 | 0.99(0.93 to 1.06) | 0.346 | 0.94(0.82 to 1.07) | 0.551 | 0.97(0.90 to 1.06) |
| Latency-associated peptide transforming growth factor beta 1 | 19 | 0.993 | 1.00(0.95 to 1.05) | 0.226 | 0.95(0.87 to 1.03) | 0.941 | 1.00(0.94 to 1.07) |
| Leukemia inhibitory factor | 5 | 0.861 | 0.99(0.86 to 1.13) | 0.481 | 1.12(0.85 to 1.48) | 0.893 | 1.01(0.88 to 1.16) |
| Macrophage colony-stimulating factor 1 | 19 | 0.492 | 0.98(0.94 to 1.03) | 0.633 | 1.03(0.91 to 1.17) | 0.622 | 0.98(0.92 to 1.05) |
| Macrophage inflammatory protein 1a | 16 | 0.223 | 1.02(0.99 to 1.06) | 0.451 | 1.02(0.97 to 1.08) | 0.106 | 1.03(0.99 to 1.07) |
| Matrix metalloproteinase-1 | 16 | 0.599 | 1.03(0.92 to 1.15) | 0.127 | 1.18(0.97 to 1.43) | 0.009 | 1.10(1.03 to 1.19) |
| Matrix metalloproteinase-10 | 16 | 0.596 | 0.99(0.96 to 1.02) | 0.835 | 1.00(0.96 to 1.05) | 0.852 | 1.00(0.96 to 1.04) |
| Monocyte chemoattractant protein-1 | 23 | 0.421 | 1.02(0.97 to 1.06) | 0.429 | 0.97(0.90 to 1.05) | 0.586 | 0.98(0.92 to 1.05) |
| Monocyte chemoattractant protein-3 | 18 | 0.910 | 1.00(0.96 to 1.04) | 0.530 | 1.03(0.94 to 1.13) | 0.553 | 1.02(0.96 to 1.08) |
| Monocyte chemoattractant protein-4 | 17 | 0.774 | 0.99(0.94 to 1.05) | 0.579 | 0.97(0.86 to 1.09) | 0.940 | 1.00(0.95 to 1.06) |
| Monocyte chemoattractant protein 2 | 4 | 0.325 | 1.06(0.94 to 1.20) | 0.662 | 1.07(0.83 to 1.37) | 0.564 | 1.05(0.90 to 1.22) |
| Natural killer cell receptor 2B4 | 21 | 0.343 | 0.98(0.93 to 1.03) | 0.901 | 0.99(0.89 to 1.10) | 0.662 | 0.99(0.94 to 1.04) |
| Neurotrophin-3 | 2 | 0.712 | 0.95(0.72 to 1.25) | NA | NA | NA | NA |
| Neurturin | 15 | 0.968 | 1.00(0.93 to 1.08) | 0.013 | 1.20(1.06 to 1.35) | 0.464 | 0.97(0.88 to 1.06) |
| Oncostatin-M | 16 | 0.977 | 1.00(0.93 to 1.08) | 0.693 | 1.04(0.85 to 1.28) | 0.199 | 0.95(0.87 to 1.03) |
| Osteoprotegerin | 21 | 0.222 | 0.97(0.93 to 1.02) | 0.547 | 1.03(0.93 to 1.14) | 0.294 | 0.97(0.91 to 1.03) |
| Programmed cell death 1 ligand 1 | 19 | 0.158 | 0.96(0.91 to 1.01) | 0.226 | 0.93(0.82 to 1.04) | 0.128 | 0.94(0.88 to 1.02) |
| Protein S100-A12 | 16 | 0.869 | 1.00(0.95 to 1.05) | 0.722 | 1.02(0.92 to 1.14) | 0.582 | 0.98(0.91 to 1.05) |
| Signaling lymphocytic activation molecule | 28 | 0.968 | 1.00(0.95 to 1.05) | 0.191 | 1.08(0.97 to 1.20) | 0.583 | 0.98(0.93 to 1.04) |
| SIR2-like protein 2 | 13 | 0.369 | 1.03(0.96 to 1.10) | 0.444 | 1.06(0.92 to 1.21) | 0.066 | 1.09(0.99 to 1.19) |
| STAM binding protein | 12 | 0.720 | 0.98(0.89 to 1.08) | 0.767 | 0.96(0.73 to 1.26) | 0.509 | 1.03(0.94 to 1.15) |
| Stem cell factor | 32 | 0.530 | 0.99(0.94 to 1.03) | 0.485 | 1.03(0.95 to 1.12) | 0.365 | 1.02(0.97 to 1.07) |
| T-cell surface glycoprotein CD5 | 23 | 0.756 | 1.01(0.96 to 1.06) | 0.207 | 0.92(0.82 to 1.04) | 0.630 | 1.02(0.95 to 1.08) |
| T-cell surface glycoprotein CD6 isoform | 16 | 0.605 | 1.01(0.98 to 1.03) | 0.342 | 1.02(0.98 to 1.05) | 0.613 | 1.01(0.98 to 1.04) |
| Thymic stromal lymphopoietin | 16 | 0.941 | 1.00(0.93 to 1.09) | 0.508 | 1.06(0.90 to 1.25) | 0.336 | 0.96(0.89 to 1.04) |
| TNF-beta | 25 | 0.164 | 1.02(0.99 to 1.05) | 0.346 | 1.02(0.98 to 1.07) | 0.612 | 1.01(0.97 to 1.04) |
| TNF-related activation-induced cytokine | 30 | 0.494 | 1.01(0.98 to 1.05) | 0.937 | 1.00(0.93 to 1.08) | 0.623 | 0.99(0.94 to 1.04) |
| TNF-related apoptosis-inducing ligand | 27 | 0.332 | 1.02(0.98 to 1.05) | 0.321 | 1.03(0.97 to 1.09) | 0.428 | 1.02(0.98 to 1.06) |
| Transforming growth factor-alpha | 14 | 0.995 | 1.00(0.92 to 1.08) | 0.091 | 0.85(0.71 to 1.01) | 0.465 | 0.97(0.89 to 1.06) |
| Tumor necrosis factor ligand superfamily member 14 | 26 | 1.000 | 1.00(0.97 to 1.03) | 0.262 | 0.97(0.92 to 1.02) | 0.838 | 0.99(0.94 to 1.05) |
| Tumor necrosis factor receptor superfamily member 9 | 28 | 0.350 | 1.02(0.98 to 1.06) | 0.711 | 1.02(0.93 to 1.12) | 0.137 | 1.04(0.99 to 1.11) |
| Urokinase-type plasminogen activator | 24 | 0.212 | 0.97(0.93 to 1.02) | 0.460 | 0.96(0.87 to 1.07) | 0.650 | 0.99(0.93 to 1.05) |
| Vascular endothelial growth factor A | 23 | 0.914 | 1.00(0.97 to 1.04) | 0.850 | 1.01(0.95 to 1.06) | 0.685 | 1.01(0.97 to 1.04) |
| NA: insufficient SNPs for MR analysis; Summary statistics of AF were obtained from the study by Nielsen JB *et al*.; All data were not adjusted for false discovery rate. | | | | | | | |

| **Table S4. MR estimates of 91 inflammatory proteins on AF** | | | | | | | |
| --- | --- | --- | --- | --- | --- | --- | --- |
| **Exposure** | **SNPs** | **Inverse variance weighted** | | **MR Egger** | | **Weighted median** | |
|  |  | *P* | OR(95%CI) | *P* | OR(95%CI) | *P* | OR(95%CI) |
| CD40L receptor levels | 15 | 0.001 | 0.93(0.89 to 0.97) | 0.044 | 0.93(0.88 to 0.99) | 0.005 | 0.93(0.89 to 0.98) |
| Fibroblast growth factor 5 levels | 23 | ＜0.001 | 1.11(1.06 to 1.16) | 0.001 | 1.14(1.06 to 1.22) | ＜0.001 | 1.15(1.10 to 1.20) |
| Leukemia inhibitory factor receptor levels | 16 | ＜0.001 | 0.86(0.80 to 0.91) | 0.005 | 0.81(0.72 to 0.92) | ＜0.001 | 0.79(0.74 to 0.86) |
| TNF-beta levels | 24 | 0.001 | 1.05(1.02 to 1.09) | 0.135 | 1.04(0.99 to 1.10) | 0.043 | 1.04(1.00 to 1.09) |
| Adenosine Deaminase levels | 14 | 0.804 | 1.01(0.94 to 1.08) | 0.755 | 0.99(0.91 to 1.07) | 0.874 | 1.00(0.96 to 1.05) |
| Artemin levels | 18 | 0.734 | 0.98(0.90 to 1.08) | 0.457 | 1.09(0.87 to 1.38) | 0.653 | 1.02(0.93 to 1.13) |
| Axin-1 levels | 8 | 0.362 | 1.06(0.94 to 1.20) | 0.035 | 1.41(1.10 to 1.81) | 0.260 | 1.09(0.94 to 1.26) |
| beta-nerve growth factor levels | 21 | 0.738 | 0.99(0.92 to 1.06) | 0.735 | 0.97(0.80 to 1.17) | 0.926 | 1.00(0.91 to 1.11) |
| C-C motif chemokine 19 levels | 20 | 0.527 | 0.98(0.92 to 1.04) | 0.281 | 0.95(0.87 to 1.04) | 0.047 | 0.93(0.87 to 1.00) |
| C-C motif chemokine 20 levels | 21 | 0.236 | 0.95(0.87 to 1.03) | 0.694 | 0.96(0.77 to 1.19) | 0.639 | 0.98(0.88 to 1.08) |
| C-C motif chemokine 23 levels | 22 | 0.693 | 1.01(0.95 to 1.08) | 0.536 | 1.03(0.94 to 1.13) | 0.805 | 1.01(0.95 to 1.06) |
| C-C motif chemokine 25 levels | 23 | 0.557 | 0.99(0.94 to 1.04) | 0.882 | 0.99(0.92 to 1.07) | 0.483 | 1.02(0.97 to 1.06) |
| C-C motif chemokine 28 levels | 19 | 0.171 | 1.12(0.95 to 1.31) | 0.459 | 1.14(0.81 to 1.59) | 0.157 | 1.09(0.97 to 1.22) |
| C-C motif chemokine 4 levels | 18 | 0.373 | 0.98(0.95 to 1.02) | 0.294 | 0.97(0.92 to 1.02) | 0.301 | 0.97(0.92 to 1.02) |
| C-X-C motif chemokine 1 levels | 9 | 0.015 | 1.08(1.02 to 1.15) | 0.199 | 1.08(0.97 to 1.19) | 0.039 | 1.07(1.00 to 1.15) |
| C-X-C motif chemokine 10 levels | 23 | 0.764 | 0.99(0.89 to 1.09) | 0.879 | 0.99(0.82 to 1.18) | 0.028 | 0.91(0.83 to 0.99) |
| C-X-C motif chemokine 11 levels | 22 | 0.014 | 1.11(1.02 to 1.20) | 0.394 | 1.10(0.89 to 1.37) | 0.046 | 1.10(1.00 to 1.22) |
| C-X-C motif chemokine 5 levels | 13 | 0.302 | 1.03(0.97 to 1.10) | 0.824 | 0.99(0.90 to 1.09) | 0.669 | 1.01(0.96 to 1.07) |
| C-X-C motif chemokine 6 levels | 9 | 0.323 | 1.05(0.95 to 1.15) | 0.613 | 0.96(0.84 to 1.10) | 0.379 | 1.02(0.97 to 1.07) |
| C-X-C motif chemokine 9 levels | 22 | 0.085 | 1.08(0.99 to 1.18) | 0.965 | 1.00(0.81 to 1.24) | 0.250 | 1.06(0.96 to 1.16) |
| Caspase 8 levels | 14 | 0.642 | 1.02(0.93 to 1.12) | 0.145 | 1.14(0.97 to 1.33) | 0.391 | 1.05(0.94 to 1.16) |
| CUB domain-containing protein 1 levels | 25 | 0.943 | 1.00(0.94 to 1.07) | 0.677 | 1.02(0.92 to 1.14) | 0.582 | 1.02(0.95 to 1.09) |
| Cystatin D levels | 23 | 0.042 | 0.95(0.90 to 1.00) | 0.211 | 0.95(0.87 to 1.03) | 0.564 | 0.99(0.94 to 1.04) |
| Delta and Notch-like epidermal growth factor-related receptor levels | 16 | 0.127 | 0.95(0.90 to 1.01) | 0.016 | 0.83(0.73 to 0.95) | 0.101 | 0.93(0.86 to 1.01) |
| Eotaxin levels | 18 | 0.091 | 1.05(0.99 to 1.12) | 0.840 | 1.01(0.90 to 1.14) | 0.345 | 1.04(0.96 to 1.13) |
| Eukaryotic translation initiation factor 4E-binding protein 1 levels | 9 | 0.308 | 1.09(0.93 to 1.27) | 0.582 | 1.12(0.77 to 1.62) | 0.155 | 1.12(0.96 to 1.31) |
| Fibroblast growth factor 19 levels | 21 | 0.508 | 0.97(0.90 to 1.06) | 0.519 | 0.93(0.76 to 1.14) | 0.585 | 0.98(0.89 to 1.07) |
| Fibroblast growth factor 21 levels | 14 | 0.878 | 1.01(0.94 to 1.08) | 0.113 | 0.88(0.76 to 1.02) | 0.665 | 0.98(0.90 to 1.07) |
| Fibroblast growth factor 23 levels | 15 | 0.973 | 1.00(0.91 to 1.10) | 0.103 | 0.79(0.60 to 1.03) | 0.447 | 0.96(0.86 to 1.07) |
| Fms-related tyrosine kinase 3 ligand levels | 29 | 0.264 | 0.97(0.91 to 1.03) | 0.814 | 0.99(0.90 to 1.09) | 0.819 | 1.01(0.94 to 1.08) |
| Fractalkine levels | 22 | 0.066 | 0.91(0.83 to 1.01) | 0.168 | 0.84(0.65 to 1.07) | 0.539 | 0.97(0.88 to 1.07) |
| Glial cell line-derived neurotrophic factor levels | 12 | 0.024 | 0.92(0.85 to 0.99) | 0.122 | 0.89(0.78 to 1.02) | 0.013 | 0.91(0.84 to 0.98) |
| Hepatocyte growth factor levels | 18 | 0.451 | 0.96(0.88 to 1.06) | 0.603 | 1.06(0.86 to 1.31) | 0.136 | 0.92(0.83 to 1.03) |
| Interferon gamma levels | 10 | 0.701 | 1.02(0.91 to 1.15) | 0.150 | 1.17(0.97 to 1.41) | 0.280 | 1.07(0.95 to 1.20) |
| Interleukin-1-alpha levels | 12 | 0.281 | 0.94(0.84 to 1.05) | 0.221 | 0.87(0.70 to 1.08) | 0.044 | 0.88(0.78 to 1.00) |
| Interleukin-10 levels | 18 | 0.060 | 0.93(0.87 to 1.00) | 0.421 | 0.94(0.80 to 1.09) | 0.038 | 0.91(0.82 to 0.99) |
| Interleukin-10 receptor subunit alpha levels | 10 | 0.597 | 0.97(0.87 to 1.08) | 0.739 | 1.03(0.87 to 1.22) | 0.941 | 1.00(0.89 to 1.13) |
| Interleukin-10 receptor subunit beta levels | 19 | 0.539 | 1.01(0.97 to 1.06) | 0.417 | 1.03(0.96 to 1.10) | 0.462 | 1.02(0.97 to 1.06) |
| Interleukin-12 subunit beta levels | 25 | 0.775 | 0.99(0.95 to 1.04) | 0.169 | 0.95(0.88 to 1.02) | 0.065 | 0.96(0.93 to 1.00) |
| Interleukin-13 levels | 11 | 0.619 | 1.03(0.93 to 1.14) | 0.360 | 1.11(0.90 to 1.37) | 0.364 | 0.95(0.85 to 1.06) |
| Interleukin-15 receptor subunit alpha levels | 12 | 0.843 | 1.01(0.93 to 1.10) | 0.262 | 0.92(0.81 to 1.05) | 0.710 | 0.99(0.95 to 1.04) |
| Interleukin-17A levels | 10 | 0.817 | 1.01(0.91 to 1.13) | 0.303 | 1.18(0.88 to 1.57) | 0.782 | 0.98(0.86 to 1.12) |
| Interleukin-17C levels | 17 | 0.957 | 1.00(0.93 to 1.08) | 0.048 | 0.79(0.64 to 0.98) | 0.251 | 1.06(0.96 to 1.17) |
| Interleukin-18 levels | 21 | 0.942 | 1.00(0.93 to 1.07) | 0.330 | 0.93(0.80 to 1.07) | 0.353 | 0.96(0.89 to 1.04) |
| interleukin-18 receptor 1 levels | 21 | 0.845 | 1.00(0.96 to 1.04) | 0.780 | 1.01(0.95 to 1.08) | 0.881 | 1.00(0.96 to 1.04) |
| Interleukin-2 levels | 15 | 0.686 | 1.02(0.94 to 1.09) | 0.597 | 0.95(0.78 to 1.15) | 0.746 | 0.98(0.90 to 1.08) |
| Interleukin-2 receptor subunit beta levels | 12 | 0.201 | 1.07(0.96 to 1.20) | 0.476 | 1.10(0.86 to 1.40) | 0.524 | 1.04(0.92 to 1.18) |
| Interleukin-20 levels | 6 | 0.174 | 1.09(0.96 to 1.22) | 0.832 | 0.98(0.79 to 1.21) | 0.210 | 1.10(0.95 to 1.27) |
| Interleukin-20 receptor subunit alpha levels | 11 | 0.353 | 1.04(0.96 to 1.12) | 0.134 | 1.15(0.97 to 1.36) | 0.089 | 1.10(0.99 to 1.22) |
| Interleukin-22 receptor subunit alpha-1 levels | 3 | 0.126 | 1.17(0.96 to 1.43) | 0.629 | 1.43(0.50 to 4.10) | 0.134 | 1.20(0.95 to 1.51) |
| Interleukin-24 levels | 10 | 0.994 | 1.00(0.89 to 1.12) | 0.859 | 1.03(0.76 to 1.38) | 0.571 | 1.04(0.90 to 1.20) |
| Interleukin-33 levels | 11 | 0.562 | 1.03(0.94 to 1.12) | 0.748 | 0.96(0.76 to 1.22) | 0.954 | 1.00(0.89 to 1.12) |
| Interleukin-4 levels | 9 | 0.649 | 1.03(0.92 to 1.15) | 0.494 | 0.90(0.68 to 1.19) | 0.606 | 1.03(0.91 to 1.18) |
| Interleukin-5 levels | 11 | 0.190 | 0.95(0.88 to 1.03) | 0.332 | 0.92(0.80 to 1.07) | 0.328 | 0.95(0.86 to 1.05) |
| Interleukin-6 levels | 2 | 0.056 | 0.85(0.72 to 1.00) | NA | NA | NA | NA |
| Interleukin-7 levels | 3 | 0.747 | 0.97(0.80 to 1.17) | 0.645 | 0.69(0.22 to 2.18) | 0.928 | 0.99(0.79 to 1.23) |
| Interleukin-8 levels | 16 | 0.928 | 1.00(0.92 to 1.08) | 0.879 | 0.99(0.84 to 1.16) | 0.460 | 0.96(0.86 to 1.07) |
| Latency-associated peptide transforming growth factor beta 1 levels | 19 | 0.202 | 1.05(0.98 to 1.13) | 0.679 | 0.97(0.86 to 1.10) | 0.831 | 1.01(0.92 to 1.11) |
| Leukemia inhibitory factor levels | 5 | 0.612 | 0.97(0.86 to 1.09) | 0.505 | 0.92(0.73 to 1.15) | 0.376 | 0.94(0.81 to 1.08) |
| Macrophage colony-stimulating factor 1 levels | 16 | 0.123 | 1.08(0.98 to 1.19) | 0.276 | 0.88(0.70 to 1.10) | 0.213 | 1.06(0.97 to 1.17) |
| Macrophage inflammatory protein 1a levels | 13 | 0.998 | 1.00(0.92 to 1.09) | 0.302 | 0.93(0.82 to 1.06) | 0.763 | 0.99(0.93 to 1.05) |
| Matrix metalloproteinase-1 levels | 16 | 0.899 | 1.01(0.91 to 1.11) | 0.224 | 1.12(0.94 to 1.33) | 0.766 | 0.99(0.89 to 1.09) |
| Matrix metalloproteinase-10 levels | 14 | 0.032 | 0.93(0.88 to 0.99) | 0.862 | 0.99(0.90 to 1.09) | 0.290 | 0.96(0.89 to 1.04) |
| Monocyte chemoattractant protein-1 levels | 21 | 0.952 | 1.00(0.95 to 1.05) | 0.661 | 0.98(0.91 to 1.06) | 0.730 | 0.99(0.93 to 1.05) |
| Monocyte chemoattractant protein-3 levels | 16 | 0.206 | 1.05(0.97 to 1.13) | 0.849 | 0.99(0.85 to 1.15) | 0.482 | 1.03(0.94 to 1.13) |
| Monocyte chemoattractant protein-4 levels | 16 | 0.895 | 1.00(0.95 to 1.06) | 0.363 | 0.95(0.86 to 1.05) | 0.687 | 0.99(0.92 to 1.06) |
| Monocyte chemoattractant protein 2 levels | 2 | 0.546 | 1.08(0.85 to 1.37) | NA | NA | NA | NA |
| Natural killer cell receptor 2B4 levels | 21 | 0.199 | 1.05(0.97 to 1.14) | 0.076 | 1.18(0.99 to 1.39) | 0.063 | 1.08(1.00 to 1.18) |
| Neurotrophin-3 levels | 2 | 0.017 | 1.34(1.05 to 1.70) | NA | NA | NA | NA |
| Neurturin levels | 14 | 0.629 | 0.98(0.89 to 1.08) | 0.657 | 1.04(0.87 to 1.24) | 0.458 | 0.96(0.87 to 1.06) |
| Oncostatin-M levels | 15 | 0.194 | 1.08(0.96 to 1.22) | 0.785 | 0.96(0.74 to 1.25) | 0.278 | 1.06(0.96 to 1.17) |
| Osteoprotegerin levels | 18 | 0.168 | 0.94(0.86 to 1.03) | 0.323 | 0.90(0.74 to 1.10) | 0.546 | 0.97(0.90 to 1.06) |
| Programmed cell death 1 ligand 1 levels | 16 | 0.80 | 1.02(0.90 to 1.15) | 0.916 | 0.98(0.72 to 1.34) | 0.668 | 1.02(0.93 to 1.13) |
| Protein S100-A12 levels | 16 | 0.331 | 1.03(0.97 to 1.11) | 0.575 | 0.96(0.84 to 1.10) | 0.439 | 1.04(0.94 to 1.14) |
| Signaling lymphocytic activation molecule levels | 25 | 0.831 | 1.01(0.94 to 1.08) | 0.416 | 1.07(0.91 to 1.25) | 0.789 | 0.99(0.91 to 1.08) |
| SIR2-like protein 2 levels | 13 | 0.587 | 0.98(0.91 to 1.06) | 0.824 | 0.98(0.83 to 1.15) | 0.739 | 1.02(0.92 to 1.13) |
| STAM binding protein levels | 11 | 0.99 | 1.00(0.91 to 1.10) | 0.947 | 1.01(0.75 to 1.36) | 0.348 | 1.07(0.93 to 1.22) |
| Stem cell factor levels | 32 | 0.869 | 1.00(0.95 to 1.04) | 0.264 | 1.05(0.97 to 1.13) | 0.786 | 0.99(0.93 to 1.05) |
| Sulfotransferase 1A1 levels | 23 | 0.47 | 0.98(0.94 to 1.03) | 0.271 | 0.94(0.84 to 1.05) | 0.801 | 0.99(0.92 to 1.06) |
| T-cell surface glycoprotein CD5 levels | 22 | 0.249 | 1.05(0.96 to 1.15) | 0.142 | 1.21(0.95 to 1.54) | 0.181 | 1.07(0.97 to 1.18) |
| T-cell surface glycoprotein CD6 isoform levels | 16 | 0.109 | 1.05(0.99 to 1.12) | 0.717 | 0.99(0.92 to 1.06) | 0.750 | 1.01(0.96 to 1.05) |
| Thymic stromal lymphopoietin levels | 15 | 0.171 | 1.06(0.97 to 1.15) | 0.098 | 1.18(0.98 to 1.42) | 0.183 | 1.08(0.97 to 1.20) |
| TNF-related activation-induced cytokine levels | 27 | 0.269 | 1.03(0.98 to 1.09) | 0.650 | 1.02(0.92 to 1.14) | 0.373 | 1.03(0.97 to 1.10) |
| TNF-related apoptosis-inducing ligand levels | 27 | 0.608 | 1.02(0.96 to 1.08) | 0.413 | 0.96(0.88 to 1.05) | 0.361 | 1.03(0.97 to 1.09) |
| Transforming growth factor-alpha levels | 14 | 0.973 | 1.00(0.87 to 1.15) | 0.050 | 0.75(0.57 to 0.97) | 0.318 | 0.94(0.84 to 1.06) |
| Tumor necrosis factor levels | 18 | 0.349 | 1.04(0.96 to 1.12) | 0.436 | 1.06(0.92 to 1.22) | 0.467 | 1.04(0.94 to 1.14) |
| Tumor necrosis factor ligand superfamily member 12 levels | 26 | 0.303 | 0.95(0.87 to 1.04) | 0.437 | 1.08(0.89 to 1.32) | 0.852 | 0.99(0.92 to 1.08) |
| Tumor necrosis factor ligand superfamily member 14 levels | 25 | 0.991 | 1.00(0.94 to 1.06) | 0.065 | 0.92(0.84 to 1.00) | 0.343 | 0.97(0.90 to 1.04) |
| Tumor necrosis factor receptor superfamily member 9 levels | 28 | 0.411 | 1.03(0.96 to 1.11) | 0.732 | 0.97(0.82 to 1.15) | 0.099 | 1.06(0.99 to 1.14) |
| Urokinase-type plasminogen activator levels | 21 | 0.678 | 0.98(0.90 to 1.07) | 0.433 | 0.93(0.78 to 1.11) | 0.269 | 0.96(0.89 to 1.03) |
| Vascular endothelial growth factor A levels | 20 | 0.695 | 1.01(0.96 to 1.06) | 0.104 | 0.94(0.88 to 1.01) | 0.515 | 0.98(0.94 to 1.03) |
| NA: insufficient SNPs for MR analysis. Summary statistics of AF were obtained from the FinnGen study; All data were not adjusted for false discovery rate. | | | | | | | |

| **Table S5. MR estimates of AF on** **inflammatory proteins** | | | | | | |
| --- | --- | --- | --- | --- | --- | --- |
| **Outcomes** | **Inverse variance weighted** | | **MR Egger** | | **Weighted median** | |
|  | *P* | OR(95%CI) | *P* | OR(95%CI) | *P* | OR(95%CI) |
| Eukaryotic translation initiation factor 4E-binding protein 1 | 0.331 | 0.98(0.95 to 1.02) | 0.326 | 0.96(0.89 to 1.04) | 0.030 | 1.00(0.94 to 1.06) |
| Adenosine Deaminase | 0.651 | 1.01(0.97 to 1.05) | 0.555 | 0.98(0.91 to 1.05) | 0.031 | 0.99(0.93 to 1.05) |
| Artemin | 0.874 | 1.00(0.96 to 1.04) | 0.514 | 0.97(0.90 to 1.05) | 0.034 | 0.97(0.91 to 1.04) |
| Axin-1 | 0.524 | 0.99(0.95 to 1.03) | 0.199 | 0.95(0.87 to 1.03) | 0.033 | 0.94(0.88 to 1.00) |
| beta-nerve growth factor | 0.421 | 1.01(0.98 to 1.05) | 0.762 | 1.01(0.94 to 1.09) | 0.030 | 1.00(0.94 to 1.06) |
| Caspase 8 | 0.714 | 0.99(0.96 to 1.03) | 0.512 | 0.98(0.90 to 1.05) | 0.034 | 0.99(0.93 to 1.06) |
| Eotaxin | 0.357 | 1.02(0.98 to 1.06) | 0.580 | 1.02(0.95 to 1.11) | 0.031 | 0.97(0.92 to 1.03) |
| C-C motif chemokine 19 | 0.799 | 1.00(0.96 to 1.03) | 0.633 | 0.98(0.91 to 1.06) | 0.036 | 0.97(0.91 to 1.04) |
| C-C motif chemokine 20 | 0.494 | 0.99(0.95 to 1.02) | 0.809 | 0.99(0.92 to 1.07) | 0.035 | 0.98(0.92 to 1.05) |
| C-C motif chemokine 23 | 0.682 | 1.01(0.97 to 1.05) | 0.148 | 1.06(0.98 to 1.14) | 0.033 | 1.04(0.98 to 1.11) |
| C-C motif chemokine 25 | 0.883 | 1.00(0.96 to 1.04) | 0.853 | 1.01(0.93 to 1.09) | 0.032 | 1.01(0.95 to 1.07) |
| C-C motif chemokine 28 | 0.994 | 1.00(0.96 to 1.04) | 0.479 | 1.03(0.95 to 1.11) | 0.035 | 1.00(0.94 to 1.07) |
| C-C motif chemokine 4 | 0.923 | 1.00(0.97 to 1.04) | 0.171 | 0.95(0.88 to 1.02) | 0.033 | 0.97(0.91 to 1.03) |
| Natural killer cell receptor 2B4 | 0.554 | 0.99(0.95 to 1.03) | 0.339 | 0.96(0.90 to 1.04) | 0.032 | 0.97(0.91 to 1.03) |
| CD40L receptor | 0.737 | 1.01(0.97 to 1.04) | 0.922 | 1.00(0.93 to 1.08) | 0.031 | 0.97(0.91 to 1.03) |
| T-cell surface glycoprotein CD5 | 0.334 | 0.98(0.95 to 1.02) | 0.380 | 0.97(0.90 to 1.04) | 0.030 | 0.94(0.89 to 1.00) |
| T-cell surface glycoprotein CD6 isoform | 0.839 | 1.00(0.96 to 1.04) | 0.582 | 0.98(0.90 to 1.06) | 0.034 | 0.97(0.90 to 1.03) |
| CUB domain-containing protein 1 | 0.758 | 0.99(0.96 to 1.03) | 0.617 | 0.98(0.91 to 1.06) | 0.035 | 0.98(0.92 to 1.05) |
| Macrophage colony-stimulating factor 1 | 0.494 | 0.99(0.95 to 1.02) | 0.939 | 1.00(0.93 to 1.08) | 0.035 | 0.99(0.93 to 1.06) |
| Cystatin D | 0.837 | 1.00(0.96 to 1.03) | 0.449 | 1.03(0.96 to 1.11) | 0.035 | 1.00(0.93 to 1.07) |
| Fractalkine | 0.882 | 1.00(0.96 to 1.04) | 0.383 | 1.04(0.95 to 1.13) | 0.031 | 1.04(0.98 to 1.10) |
| C-X-C motif chemokine 1 | 0.596 | 0.99(0.95 to 1.03) | 0.238 | 0.95(0.87 to 1.03) | 0.034 | 0.96(0.89 to 1.02) |
| C-X-C motif chemokine 10 | 0.886 | 1.00(0.97 to 1.04) | 0.395 | 0.97(0.90 to 1.04) | 0.034 | 0.97(0.91 to 1.04) |
| C-X-C motif chemokine 11 | 0.860 | 1.00(0.96 to 1.05) | 0.271 | 0.95(0.87 to 1.04) | 0.031 | 0.98(0.92 to 1.04) |
| C-X-C motif chemokine 5 | 0.549 | 0.98(0.92 to 1.04) | 0.426 | 0.95(0.84 to 1.08) | 0.036 | 0.97(0.91 to 1.04) |
| C-X-C motif chemokine 6 | 0.375 | 0.98(0.94 to 1.02) | 0.105 | 0.93(0.86 to 1.01) | 0.030 | 0.95(0.90 to 1.01) |
| C-X-C motif chemokine 9 | 0.213 | 0.98(0.94 to 1.01) | 0.208 | 0.95(0.89 to 1.03) | 0.033 | 0.97(0.91 to 1.03) |
| Delta and Notch-like epidermal growth factor-related receptor | 0.257 | 1.02(0.98 to 1.06) | 0.357 | 1.04(0.96 to 1.12) | 0.031 | 1.05(0.99 to 1.12) |
| Protein S100-A12 | 0.851 | 1.00(0.96 to 1.04) | 0.937 | 1.00(0.93 to 1.09) | 0.034 | 1.03(0.97 to 1.10) |
| Fibroblast growth factor 19 | 0.890 | 1.00(0.95 to 1.04) | 0.734 | 0.98(0.90 to 1.08) | 0.035 | 0.96(0.89 to 1.03) |
| Fibroblast growth factor 21 | 0.657 | 0.99(0.95 to 1.03) | 0.607 | 1.02(0.94 to 1.11) | 0.036 | 1.00(0.93 to 1.07) |
| Fibroblast growth factor 23 | 0.790 | 1.00(0.97 to 1.04) | 0.601 | 0.98(0.91 to 1.06) | 0.031 | 0.96(0.91 to 1.03) |
| Fibroblast growth factor 5 | 0.453 | 1.06(0.90 to 1.25) | 0.644 | 0.93(0.68 to 1.27) | 0.034 | 0.99(0.92 to 1.06) |
| Fms-related tyrosine kinase 3 ligand | 0.436 | 0.98(0.94 to 1.03) | 0.753 | 1.01(0.93 to 1.11) | 0.030 | 1.01(0.96 to 1.08) |
| Glial cell line-derived neurotrophic factor | 0.354 | 1.02(0.98 to 1.06) | 0.742 | 1.01(0.94 to 1.09) | 0.034 | 1.00(0.93 to 1.06) |
| Hepatocyte growth factor | 0.601 | 0.99(0.96 to 1.03) | 0.447 | 1.03(0.96 to 1.11) | 0.032 | 1.02(0.95 to 1.08) |
| Interferon gamma | 0.284 | 1.02(0.98 to 1.06) | 0.043 | 0.92(0.86 to 1.00) | 0.032 | 1.00(0.94 to 1.07) |
| Interleukin-10 | 0.398 | 0.98(0.95 to 1.02) | 0.164 | 0.95(0.88 to 1.02) | 0.034 | 0.98(0.92 to 1.04) |
| Interleukin-10 receptor subunit alpha | 0.931 | 1.00(0.96 to 1.05) | 0.267 | 0.95(0.88 to 1.04) | 0.032 | 0.97(0.91 to 1.03) |
| Interleukin-10 receptor subunit beta | 0.207 | 0.98(0.94 to 1.01) | 0.950 | 1.00(0.93 to 1.08) | 0.031 | 0.95(0.89 to 1.01) |
| Interleukin-12 subunit beta | 0.201 | 0.98(0.94 to 1.01) | 0.022 | 0.92(0.85 to 0.99) | 0.030 | 0.94(0.88 to 0.99) |
| Interleukin-13 | 0.415 | 0.98(0.94 to 1.03) | 0.943 | 1.00(0.92 to 1.09) | 0.036 | 0.97(0.90 to 1.04) |
| Interleukin-15 receptor subunit alpha | 0.591 | 0.99(0.95 to 1.03) | 0.213 | 0.95(0.88 to 1.03) | 0.036 | 0.97(0.90 to 1.04) |
| Interleukin-17A | 0.906 | 1.00(0.96 to 1.05) | 0.364 | 0.96(0.89 to 1.04) | 0.034 | 0.97(0.90 to 1.03) |
| Interleukin-17C | 0.758 | 0.99(0.95 to 1.04) | 0.037 | 0.92(0.85 to 0.99) | 0.034 | 0.96(0.90 to 1.02) |
| Interleukin-18 | 0.591 | 0.99(0.95 to 1.03) | 0.547 | 0.98(0.91 to 1.05) | 0.031 | 0.96(0.91 to 1.02) |
| interleukin-18 receptor 1 | 0.728 | 0.99(0.96 to 1.03) | 0.791 | 1.01(0.94 to 1.09) | 0.033 | 0.98(0.92 to 1.05) |
| Interleukin-1-alpha | 0.673 | 1.01(0.97 to 1.05) | 0.507 | 1.03(0.95 to 1.11) | 0.034 | 1.04(0.98 to 1.12) |
| Interleukin-2 | 0.939 | 1.00(0.96 to 1.05) | 0.909 | 1.00(0.91 to 1.08) | 0.037 | 1.00(0.93 to 1.07) |
| Interleukin-20 | 0.424 | 0.98(0.95 to 1.02) | 0.190 | 0.95(0.88 to 1.03) | 0.036 | 0.97(0.90 to 1.04) |
| Interleukin-20 receptor subunit alpha | 0.980 | 1.00(0.96 to 1.04) | 0.182 | 0.95(0.88 to 1.02) | 0.034 | 0.95(0.88 to 1.01) |
| Interleukin-22 receptor subunit alpha-1 | 0.593 | 0.99(0.95 to 1.03) | 0.346 | 0.96(0.89 to 1.04) | 0.034 | 0.95(0.89 to 1.01) |
| Interleukin-24 | 0.949 | 1.00(0.96 to 1.04) | 0.537 | 0.98(0.90 to 1.06) | 0.039 | 1.00(0.92 to 1.08) |
| Interleukin-2 receptor subunit beta | 0.317 | 0.98(0.94 to 1.02) | 0.094 | 0.94(0.87 to 1.01) | 0.037 | 0.95(0.88 to 1.02) |
| Interleukin-33 | 0.826 | 1.00(0.96 to 1.05) | 0.833 | 0.99(0.91 to 1.08) | 0.038 | 1.01(0.94 to 1.09) |
| Interleukin-4 | 0.934 | 1.00(0.96 to 1.05) | 0.619 | 1.02(0.94 to 1.11) | 0.038 | 0.98(0.91 to 1.06) |
| Interleukin-5 | 0.377 | 0.98(0.94 to 1.02) | 0.339 | 0.96(0.89 to 1.04) | 0.032 | 0.97(0.91 to 1.03) |
| Interleukin-6 | 0.397 | 0.98(0.93 to 1.03) | 0.427 | 1.04(0.94 to 1.15) | 0.033 | 1.01(0.94 to 1.08) |
| Interleukin-7 | 0.238 | 0.98(0.94 to 1.02) | 0.055 | 0.92(0.85 to 1.00) | 0.036 | 0.94(0.88 to 1.01) |
| Interleukin-8 | 0.903 | 1.00(0.96 to 1.04) | 0.964 | 1.00(0.93 to 1.08) | 0.031 | 1.00(0.94 to 1.06) |
| Latency-associated peptide transforming growth factor beta 1 | 0.456 | 1.01(0.98 to 1.06) | 0.783 | 1.01(0.93 to 1.09) | 0.032 | 0.98(0.92 to 1.05) |
| Leukemia inhibitory factor | 0.336 | 0.98(0.94 to 1.02) | 0.831 | 0.99(0.91 to 1.07) | 0.037 | 0.99(0.92 to 1.07) |
| Leukemia inhibitory factor receptor | 0.007 | 1.06(1.02 to 1.10) | 0.093 | 1.07(0.99 to 1.16) | 0.031 | 1.11(1.04 to 1.18) |
| Monocyte chemoattractant protein-1 | 0.882 | 1.00(0.97 to 1.04) | 0.350 | 1.04(0.96 to 1.12) | 0.035 | 1.01(0.94 to 1.08) |
| Monocyte chemoattractant protein 2 | 0.957 | 1.00(0.96 to 1.04) | 0.890 | 0.99(0.92 to 1.07) | 0.035 | 1.02(0.95 to 1.09) |
| Monocyte chemoattractant protein-3 | 0.466 | 0.99(0.95 to 1.03) | 0.626 | 0.98(0.91 to 1.06) | 0.034 | 0.94(0.88 to 1.01) |
| Monocyte chemoattractant protein-4 | 0.938 | 1.00(0.96 to 1.04) | 0.970 | 1.00(0.93 to 1.08) | 0.034 | 0.97(0.91 to 1.03) |
| Macrophage inflammatory protein 1a | 0.980 | 1.00(0.96 to 1.04) | 0.718 | 0.99(0.91 to 1.07) | 0.031 | 0.97(0.91 to 1.03) |
| Matrix metalloproteinase-1 | 0.987 | 1.00(0.96 to 1.05) | 0.422 | 1.04(0.95 to 1.13) | 0.036 | 0.98(0.92 to 1.06) |
| Matrix metalloproteinase-10 | 0.192 | 0.97(0.94 to 1.01) | 0.861 | 1.01(0.93 to 1.09) | 0.037 | 0.99(0.92 to 1.06) |
| Neurturin | 0.288 | 0.98(0.94 to 1.02) | 0.932 | 1.00(0.93 to 1.09) | 0.037 | 1.01(0.94 to 1.08) |
| Neurotrophin-3 | 0.720 | 0.99(0.96 to 1.03) | 0.703 | 1.02(0.94 to 1.10) | 0.030 | 0.98(0.93 to 1.04) |
| Osteoprotegerin | 0.108 | 1.03(0.99 to 1.07) | 0.112 | 1.06(0.99 to 1.14) | 0.030 | 1.07(1.01 to 1.13) |
| Oncostatin-M | 0.393 | 0.98(0.95 to 1.02) | 0.369 | 0.96(0.89 to 1.04) | 0.031 | 0.97(0.92 to 1.03) |
| Programmed cell death 1 ligand 1 | 0.816 | 1.00(0.96 to 1.03) | 0.989 | 1.00(0.93 to 1.08) | 0.034 | 0.99(0.93 to 1.06) |
| Stem cell factor | 0.009 | 0.95(0.92 to 0.99) | 0.132 | 0.94(0.87 to 1.02) | 0.033 | 0.93(0.87 to 0.99) |
| SIR2-like protein 2 | 0.718 | 0.99(0.95 to 1.03) | 0.210 | 0.95(0.88 to 1.03) | 0.031 | 0.94(0.88 to 1.00) |
| Signaling lymphocytic activation molecule | 0.186 | 1.03(0.99 to 1.06) | 0.798 | 1.01(0.94 to 1.09) | 0.037 | 1.04(0.96 to 1.11) |
| Sulfotransferase 1A1 | 0.430 | 0.98(0.94 to 1.02) | 0.151 | 0.94(0.87 to 1.02) | 0.034 | 0.93(0.87 to 1.00) |
| STAM binding protein | 0.785 | 0.99(0.96 to 1.03) | 0.373 | 0.97(0.90 to 1.04) | 0.031 | 0.96(0.91 to 1.02) |
| Transforming growth factor-alpha | 0.619 | 1.01(0.97 to 1.05) | 0.502 | 1.03(0.95 to 1.11) | 0.035 | 0.99(0.93 to 1.06) |
| Tumor necrosis factor | 0.215 | 1.03(0.99 to 1.07) | 0.321 | 1.04(0.96 to 1.12) | 0.037 | 1.05(0.98 to 1.13) |
| TNF-beta | 0.105 | 1.03(0.99 to 1.08) | 0.750 | 1.01(0.94 to 1.10) | 0.036 | 0.99(0.92 to 1.06) |
| Tumor necrosis factor receptor superfamily member 9 | 0.068 | 0.96(0.92 to 1.00) | 0.072 | 0.93(0.86 to 1.01) | 0.033 | 0.94(0.88 to 1.00) |
| Tumor necrosis factor ligand superfamily member 14 | 0.859 | 1.00(0.96 to 1.04) | 0.816 | 0.99(0.92 to 1.07) | 0.036 | 0.98(0.92 to 1.06) |
| TNF-related apoptosis-inducing ligand | 0.141 | 0.97(0.94 to 1.01) | 0.230 | 0.96(0.89 to 1.03) | 0.036 | 0.98(0.92 to 1.05) |
| TNF-related activation-induced cytokine | 0.157 | 0.97(0.93 to 1.01) | 0.188 | 0.94(0.87 to 1.03) | 0.032 | 0.93(0.87 to 0.99) |
| Thymic stromal lymphopoietin | 0.711 | 1.01(0.96 to 1.06) | 0.530 | 1.03(0.94 to 1.13) | 0.036 | 1.04(0.97 to 1.12) |
| Tumor necrosis factor ligand superfamily member 12 | 0.828 | 1.00(0.96 to 1.03) | 0.645 | 0.98(0.91 to 1.06) | 0.031 | 0.98(0.92 to 1.04) |
| Urokinase-type plasminogen activator | 0.663 | 0.99(0.96 to 1.03) | 0.588 | 0.98(0.91 to 1.05) | 0.033 | 0.96(0.90 to 1.03) |
| Vascular endothelial growth factor A | 0.814 | 0.99(0.95 to 1.04) | 0.945 | 1.00(0.91 to 1.09) | 0.030 | 0.96(0.90 to 1.02) |
| Summary statistics of AF were obtained from the study by Nielsen JB *et al*. ; All data were not adjusted for false discovery rate. | | | | | | |

| **Table S6. MR estimates of AF on** **inflammatory proteins** | | | | | | |
| --- | --- | --- | --- | --- | --- | --- |
| **Outcomes** | **Inverse variance weighted** | | **MR Egger** | | **Weighted median** | |
|  | *P* | OR(95%CI) | *P* | OR(95%CI) | *P* | OR(95%CI) |
| Eukaryotic translation initiation factor 4E-binding protein 1 | 0.944 | 1.00(0.97 to 1.04) | 0.751 | 1.01(0.94 to 1.09) | 0.646 | 1.01(0.95 to 1.08) |
| Adenosine Deaminase | 0.528 | 0.99(0.95 to 1.02) | 0.427 | 0.97(0.90 to 1.05) | 0.042 | 0.95(0.90 to 1.00) |
| Artemin | 0.086 | 0.96(0.92 to 1.01) | 0.608 | 1.02(0.94 to 1.12) | 0.655 | 0.98(0.92 to 1.05) |
| Axin-1 | 0.410 | 0.98(0.95 to 1.02) | 0.753 | 1.01(0.93 to 1.10) | 0.384 | 0.97(0.91 to 1.04) |
| beta-nerve growth factor | 0.573 | 1.01(0.98 to 1.04) | 0.314 | 0.96(0.90 to 1.04) | 0.726 | 0.99(0.94 to 1.05) |
| Caspase 8 | 0.211 | 0.98(0.95 to 1.01) | 0.817 | 1.01(0.94 to 1.08) | 0.980 | 1.00(0.95 to 1.06) |
| Eotaxin | 0.551 | 1.01(0.98 to 1.04) | 0.627 | 1.02(0.95 to 1.09) | 0.901 | 1.00(0.94 to 1.07) |
| C-C motif chemokine 19 | 0.730 | 0.99(0.96 to 1.03) | 0.801 | 1.01(0.93 to 1.09) | 0.530 | 1.02(0.96 to 1.08) |
| C-C motif chemokine 20 | 0.888 | 1.00(0.96 to 1.03) | 0.688 | 0.98(0.91 to 1.06) | 0.943 | 1.00(0.95 to 1.06) |
| C-C motif chemokine 23 | 0.195 | 0.98(0.95 to 1.01) | 0.687 | 0.99(0.92 to 1.06) | 0.379 | 0.97(0.92 to 1.03) |
| C-C motif chemokine 25 | 0.169 | 0.96(0.90 to 1.02) | 0.831 | 1.01(0.89 to 1.15) | 0.700 | 0.99(0.93 to 1.05) |
| C-C motif chemokine 28 | 0.584 | 0.99(0.96 to 1.03) | 0.376 | 1.03(0.96 to 1.12) | 0.751 | 0.99(0.94 to 1.04) |
| C-C motif chemokine 4 | 0.060 | 0.97(0.93 to 1.00) | 0.539 | 0.98(0.90 to 1.05) | 0.226 | 0.96(0.91 to 1.02) |
| Natural killer cell receptor 2B4 | 0.304 | 0.98(0.95 to 1.02) | 0.534 | 0.98(0.91 to 1.05) | 0.329 | 0.97(0.92 to 1.03) |
| CD40L receptor | 0.614 | 0.99(0.96 to 1.02) | 0.785 | 1.01(0.94 to 1.08) | 0.553 | 0.98(0.93 to 1.04) |
| T-cell surface glycoprotein CD5 | 0.914 | 1.00(0.97 to 1.03) | 0.520 | 0.98(0.91 to 1.05) | 0.096 | 0.96(0.91 to 1.01) |
| T-cell surface glycoprotein CD6 isoform | 0.308 | 1.02(0.98 to 1.06) | 0.769 | 0.99(0.91 to 1.07) | 0.262 | 0.97(0.92 to 1.02) |
| CUB domain-containing protein 1 | 0.986 | 1.00(0.97 to 1.03) | 0.263 | 0.96(0.90 to 1.03) | 0.906 | 1.00(0.95 to 1.06) |
| Macrophage colony-stimulating factor 1 | 0.292 | 0.98(0.95 to 1.02) | 0.317 | 0.96(0.89 to 1.04) | 0.405 | 0.98(0.92 to 1.03) |
| Cystatin D | 0.628 | 1.01(0.97 to 1.04) | 0.624 | 1.02(0.95 to 1.10) | 0.850 | 1.01(0.95 to 1.07) |
| Fractalkine | 0.651 | 0.99(0.95 to 1.03) | 0.552 | 1.03(0.94 to 1.13) | 0.316 | 1.03(0.97 to 1.09) |
| C-X-C motif chemokine 1 | 0.117 | 0.97(0.94 to 1.01) | 0.412 | 0.97(0.90 to 1.04) | 0.170 | 0.96(0.91 to 1.02) |
| C-X-C motif chemokine 10 | 0.933 | 1.00(0.96 to 1.04) | 0.205 | 0.95(0.88 to 1.03) | 0.843 | 0.99(0.94 to 1.06) |
| C-X-C motif chemokine 11 | 0.256 | 0.98(0.94 to 1.02) | 0.553 | 0.98(0.90 to 1.06) | 0.392 | 0.97(0.92 to 1.03) |
| C-X-C motif chemokine 5 | 0.101 | 0.97(0.94 to 1.01) | 0.408 | 0.97(0.90 to 1.04) | 0.122 | 0.95(0.90 to 1.01) |
| C-X-C motif chemokine 6 | 0.007 | 0.95(0.92 to 0.99) | 0.128 | 0.94(0.87 to 1.02) | 0.064 | 0.94(0.87 to 1.00) |
| C-X-C motif chemokine 9 | 0.060 | 0.97(0.94 to 1.00) | 0.028 | 0.92(0.86 to 0.99) | 0.444 | 0.98(0.92 to 1.03) |
| Delta and Notch-like epidermal growth factor-related receptor | 0.592 | 1.01(0.98 to 1.04) | 0.655 | 1.02(0.95 to 1.09) | 0.922 | 1.00(0.95 to 1.06) |
| Protein S100-A12 | 0.851 | 1.00(0.96 to 1.03) | 0.919 | 1.00(0.93 to 1.07) | 0.494 | 1.02(0.96 to 1.08) |
| Fibroblast growth factor 19 | 0.478 | 0.99(0.95 to 1.02) | 0.952 | 1.00(0.93 to 1.07) | 0.193 | 0.96(0.91 to 1.02) |
| Fibroblast growth factor 21 | 0.857 | 1.00(0.97 to 1.04) | 0.517 | 1.03(0.95 to 1.11) | 0.548 | 0.98(0.92 to 1.04) |
| Fibroblast growth factor 23 | 0.347 | 1.02(0.98 to 1.05) | 0.096 | 0.94(0.88 to 1.01) | 0.489 | 0.98(0.93 to 1.04) |
| Fibroblast growth factor 5 | 0.974 | 1.00(0.96 to 1.04) | 0.194 | 0.95(0.88 to 1.03) | 0.246 | 0.97(0.91 to 1.02) |
| Fms-related tyrosine kinase 3 ligand | 0.453 | 0.99(0.95 to 1.02) | 0.283 | 0.95(0.88 to 1.04) | 0.635 | 1.01(0.96 to 1.07) |
| Glial cell line-derived neurotrophic factor | 0.783 | 1.00(0.97 to 1.04) | 0.487 | 0.98(0.91 to 1.05) | 0.583 | 0.98(0.93 to 1.04) |
| Hepatocyte growth factor | 0.434 | 0.99(0.95 to 1.02) | 0.690 | 1.02(0.94 to 1.09) | 0.470 | 1.02(0.97 to 1.08) |
| Interferon gamma | 0.651 | 0.99(0.95 to 1.03) | 0.460 | 0.97(0.89 to 1.05) | 0.500 | 0.98(0.92 to 1.04) |
| Interleukin-10 | 0.403 | 0.99(0.95 to 1.02) | 0.087 | 0.94(0.87 to 1.01) | 0.130 | 0.96(0.90 to 1.01) |
| Interleukin-10 receptor subunit alpha | 0.302 | 0.98(0.94 to 1.02) | 0.322 | 0.96(0.88 to 1.04) | 0.703 | 0.99(0.93 to 1.05) |
| Interleukin-10 receptor subunit beta | 0.090 | 0.97(0.94 to 1.00) | 0.987 | 1.00(0.93 to 1.08) | 0.368 | 0.97(0.92 to 1.03) |
| Interleukin-12 subunit beta | 0.899 | 1.00(0.96 to 1.03) | 0.011 | 0.91(0.85 to 0.98) | 0.251 | 0.97(0.92 to 1.02) |
| Interleukin-13 | 0.271 | 0.98(0.94 to 1.02) | 0.890 | 0.99(0.91 to 1.08) | 0.235 | 0.96(0.90 to 1.03) |
| Interleukin-15 receptor subunit alpha | 0.151 | 0.97(0.93 to 1.01) | 0.393 | 0.96(0.89 to 1.05) | 0.191 | 0.96(0.91 to 1.02) |
| Interleukin-17A | 0.208 | 1.03(0.99 to 1.07) | 0.543 | 0.97(0.89 to 1.06) | 0.383 | 1.03(0.97 to 1.09) |
| Interleukin-17C | 0.241 | 0.98(0.94 to 1.02) | 0.780 | 0.99(0.91 to 1.07) | 0.599 | 0.98(0.93 to 1.05) |
| Interleukin-18 | 0.815 | 1.00(0.96 to 1.03) | 0.521 | 1.02(0.95 to 1.10) | 0.841 | 0.99(0.94 to 1.05) |
| interleukin-18 receptor 1 | 0.495 | 1.01(0.98 to 1.05) | 0.603 | 1.02(0.95 to 1.10) | 0.873 | 1.00(0.94 to 1.06) |
| Interleukin-1-alpha | 0.453 | 0.99(0.95 to 1.02) | 0.956 | 1.00(0.92 to 1.09) | 0.812 | 1.01(0.95 to 1.07) |
| Interleukin-2 | 0.174 | 0.97(0.94 to 1.01) | 0.863 | 1.01(0.93 to 1.09) | 0.653 | 0.99(0.92 to 1.05) |
| Interleukin-20 | 0.776 | 0.99(0.96 to 1.03) | 0.235 | 0.95(0.88 to 1.03) | 0.232 | 0.96(0.90 to 1.03) |
| Interleukin-20 receptor subunit alpha | 0.477 | 0.98(0.94 to 1.03) | 0.153 | 0.93(0.85 to 1.02) | 0.056 | 0.94(0.88 to 1.00) |
| Interleukin-22 receptor subunit alpha-1 | 0.235 | 0.98(0.94 to 1.02) | 0.452 | 0.97(0.89 to 1.05) | 0.329 | 0.97(0.91 to 1.03) |
| Interleukin-24 | 0.680 | 0.99(0.95 to 1.03) | 0.943 | 1.00(0.91 to 1.09) | 0.715 | 1.01(0.95 to 1.08) |
| Interleukin-2 receptor subunit beta | 0.046 | 0.96(0.93 to 1.00) | 0.653 | 0.98(0.90 to 1.06) | 0.410 | 0.97(0.92 to 1.04) |
| Interleukin-33 | 0.001 | 0.93(0.90 to 0.97) | 0.268 | 0.95(0.88 to 1.04) | 0.146 | 0.95(0.89 to 1.02) |
| Interleukin-4 | 0.990 | 1.00(0.96 to 1.04) | 0.594 | 1.02(0.94 to 1.11) | 0.341 | 0.97(0.91 to 1.03) |
| Interleukin-5 | 0.728 | 0.99(0.96 to 1.03) | 0.455 | 0.97(0.89 to 1.05) | 0.675 | 0.99(0.92 to 1.05) |
| Interleukin-6 | 0.596 | 1.01(0.98 to 1.04) | 0.611 | 1.02(0.95 to 1.09) | 0.645 | 1.01(0.96 to 1.08) |
| Interleukin-7 | 0.236 | 0.98(0.94 to 1.02) | 0.943 | 1.00(0.93 to 1.09) | 0.994 | 1.00(0.94 to 1.07) |
| Interleukin-8 | 0.351 | 0.98(0.95 to 1.02) | 0.248 | 0.95(0.88 to 1.03) | 0.276 | 0.97(0.92 to 1.03) |
| Latency-associated peptide transforming growth factor beta 1 | 0.924 | 1.00(0.97 to 1.04) | 0.699 | 1.02(0.94 to 1.10) | 0.896 | 1.00(0.94 to 1.06) |
| Leukemia inhibitory factor | 0.202 | 0.98(0.94 to 1.01) | 0.826 | 1.01(0.93 to 1.10) | 0.819 | 1.01(0.94 to 1.08) |
| Leukemia inhibitory factor receptor | 0.929 | 1.00(0.93 to 1.09) | 0.678 | 1.04(0.87 to 1.23) | 0.580 | 1.02(0.96 to 1.08) |
| Monocyte chemoattractant protein-1 | 0.085 | 0.97(0.94 to 1.00) | 0.335 | 0.96(0.90 to 1.04) | 0.636 | 0.99(0.93 to 1.05) |
| Monocyte chemoattractant protein 2 | 0.293 | 0.98(0.95 to 1.02) | 0.241 | 0.96(0.89 to 1.03) | 0.484 | 0.98(0.92 to 1.04) |
| Monocyte chemoattractant protein-3 | 0.972 | 1.00(0.96 to 1.04) | 0.719 | 1.02(0.93 to 1.10) | 0.871 | 1.01(0.94 to 1.08) |
| Monocyte chemoattractant protein-4 | 0.434 | 0.99(0.95 to 1.02) | 0.859 | 1.01(0.93 to 1.09) | 0.296 | 0.97(0.91 to 1.03) |
| Macrophage inflammatory protein 1a | 0.190 | 0.98(0.95 to 1.01) | 0.332 | 0.97(0.90 to 1.04) | 0.095 | 0.95(0.90 to 1.01) |
| Matrix metalloproteinase-1 | 0.185 | 0.97(0.94 to 1.01) | 0.609 | 1.02(0.94 to 1.11) | 0.492 | 0.98(0.92 to 1.04) |
| Matrix metalloproteinase-10 | 0.229 | 1.02(0.99 to 1.06) | 0.396 | 1.03(0.96 to 1.12) | 0.453 | 1.02(0.96 to 1.09) |
| Neurturin | 0.131 | 0.97(0.93 to 1.01) | 0.958 | 1.00(0.92 to 1.09) | 0.788 | 0.99(0.93 to 1.06) |
| Neurotrophin-3 | 0.370 | 1.02(0.98 to 1.05) | 0.267 | 0.96(0.89 to 1.03) | 0.862 | 1.00(0.94 to 1.05) |
| Osteoprotegerin | 0.638 | 1.01(0.98 to 1.04) | 0.388 | 0.97(0.91 to 1.04) | 0.585 | 1.02(0.96 to 1.08) |
| Oncostatin-M | 0.409 | 0.98(0.95 to 1.02) | 0.818 | 0.99(0.91 to 1.07) | 0.738 | 0.99(0.93 to 1.05) |
| Programmed cell death 1 ligand 1 | 0.353 | 0.98(0.95 to 1.02) | 0.841 | 1.01(0.93 to 1.09) | 0.798 | 0.99(0.94 to 1.05) |
| Stem cell factor | 0.394 | 0.98(0.95 to 1.02) | 0.656 | 0.98(0.91 to 1.06) | 0.921 | 1.00(0.95 to 1.06) |
| SIR2-like protein 2 | 0.607 | 0.99(0.96 to 1.03) | 0.949 | 1.00(0.93 to 1.08) | 0.613 | 0.98(0.93 to 1.05) |
| Signaling lymphocytic activation molecule | 0.220 | 1.02(0.99 to 1.06) | 0.416 | 1.03(0.96 to 1.11) | 0.397 | 1.03(0.96 to 1.09) |
| Sulfotransferase 1A1 | 0.151 | 0.97(0.93 to 1.01) | 0.935 | 1.00(0.91 to 1.09) | 0.056 | 0.94(0.89 to 1.00) |
| STAM binding protein | 0.665 | 0.99(0.96 to 1.03) | 0.656 | 1.02(0.95 to 1.09) | 0.856 | 0.99(0.94 to 1.06) |
| Transforming growth factor-alpha | 0.852 | 1.00(0.97 to 1.04) | 0.584 | 1.02(0.94 to 1.11) | 0.937 | 1.00(0.94 to 1.06) |
| Tumor necrosis factor | 0.856 | 1.00(0.96 to 1.05) | 0.301 | 1.05(0.96 to 1.15) | 0.988 | 1.00(0.94 to 1.06) |
| TNF-beta | 0.695 | 0.99(0.95 to 1.03) | 0.057 | 0.92(0.84 to 1.00) | 0.646 | 0.98(0.91 to 1.06) |
| Tumor necrosis factor receptor superfamily member 9 | 0.405 | 0.98(0.94 to 1.02) | 0.057 | 0.92(0.84 to 1.00) | 0.556 | 0.98(0.93 to 1.04) |
| Tumor necrosis factor ligand superfamily member 14 | 0.152 | 0.97(0.94 to 1.01) | 0.500 | 1.03(0.95 to 1.12) | 0.829 | 0.99(0.93 to 1.06) |
| TNF-related apoptosis-inducing ligand | 0.145 | 0.97(0.94 to 1.01) | 0.856 | 0.99(0.92 to 1.07) | 0.943 | 1.00(0.94 to 1.06) |
| TNF-related activation-induced cytokine | 0.804 | 1.00(0.96 to 1.03) | 0.540 | 0.98(0.90 to 1.05) | 0.530 | 0.98(0.93 to 1.04) |
| Thymic stromal lymphopoietin | 0.912 | 1.00(0.96 to 1.04) | 0.510 | 1.03(0.94 to 1.13) | 0.562 | 1.02(0.95 to 1.09) |
| Tumor necrosis factor ligand superfamily member 12 | 0.234 | 0.97(0.93 to 1.02) | 0.688 | 0.98(0.89 to 1.08) | 0.604 | 0.98(0.93 to 1.05) |
| Urokinase-type plasminogen activator | 0.921 | 1.00(0.96 to 1.04) | 0.172 | 0.95(0.87 to 1.02) | 0.490 | 0.98(0.92 to 1.04) |
| Vascular endothelial growth factor A | 0.164 | 0.98(0.94 to 1.01) | 0.414 | 0.97(0.90 to 1.04) | 0.177 | 0.96(0.90 to 1.02) |
| Summary statistics of AF were obtained from the FinnGen study; All data were not adjusted for false discovery rate. | | | | | | |

**Figure S1**

**
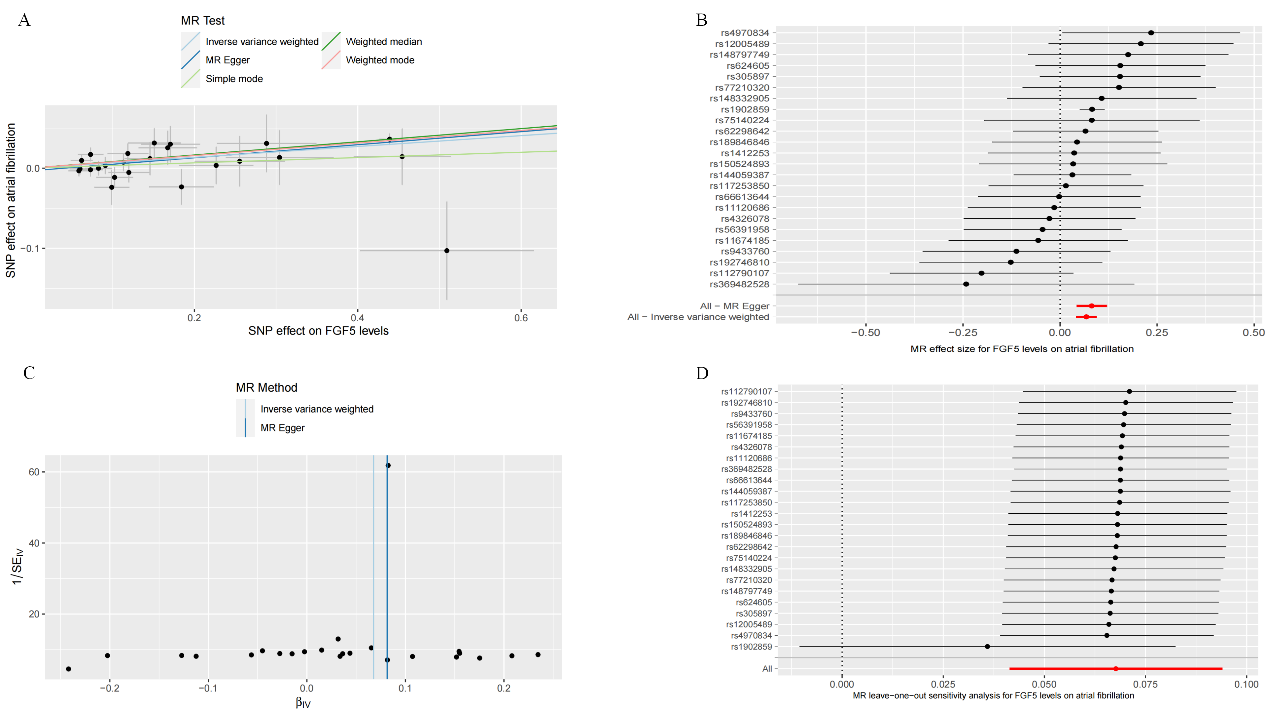
**

**Figure S1.** Mendelian randomization (MR) analysis of fibroblast growth factor 5 and AF, when GWAS of AF from the study by Nielsen JB *et al.* were used as outcome. (A) Scatter plot of MR analysis; (B) Forest plot of MR analysis; (C) Funnel plots of MR analysis; (D) leave-one-out sensitivity analysis of MR analysis.

**Figure S2**

**
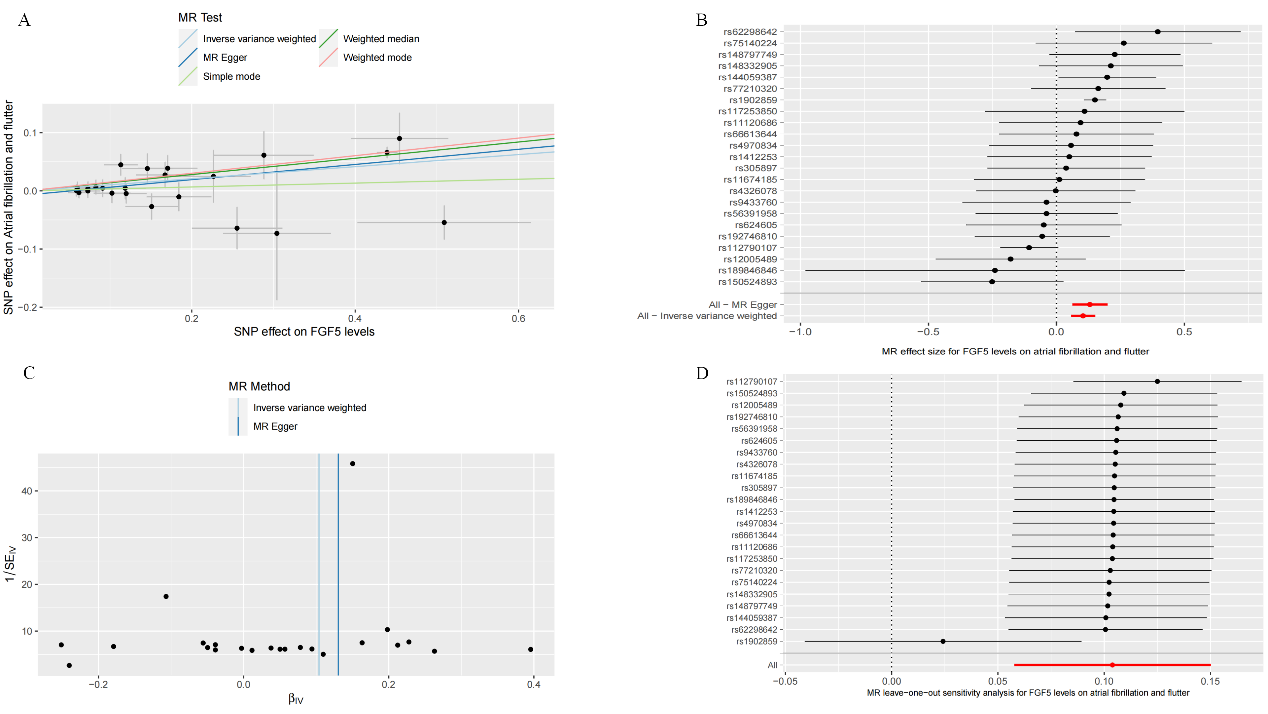
**

**Figure S2.** Mendelian randomization (MR) analysis of fibroblast growth factor 5 and AF, when GWAS of AF from the FinnGen study were used as outcome. (A) Scatter plot of MR analysis; (B) Forest plot of MR analysis; (C) Funnel plots of MR analysis; (D) leave-one-out sensitivity analysis of MR analysis.

**Figure S3**

**
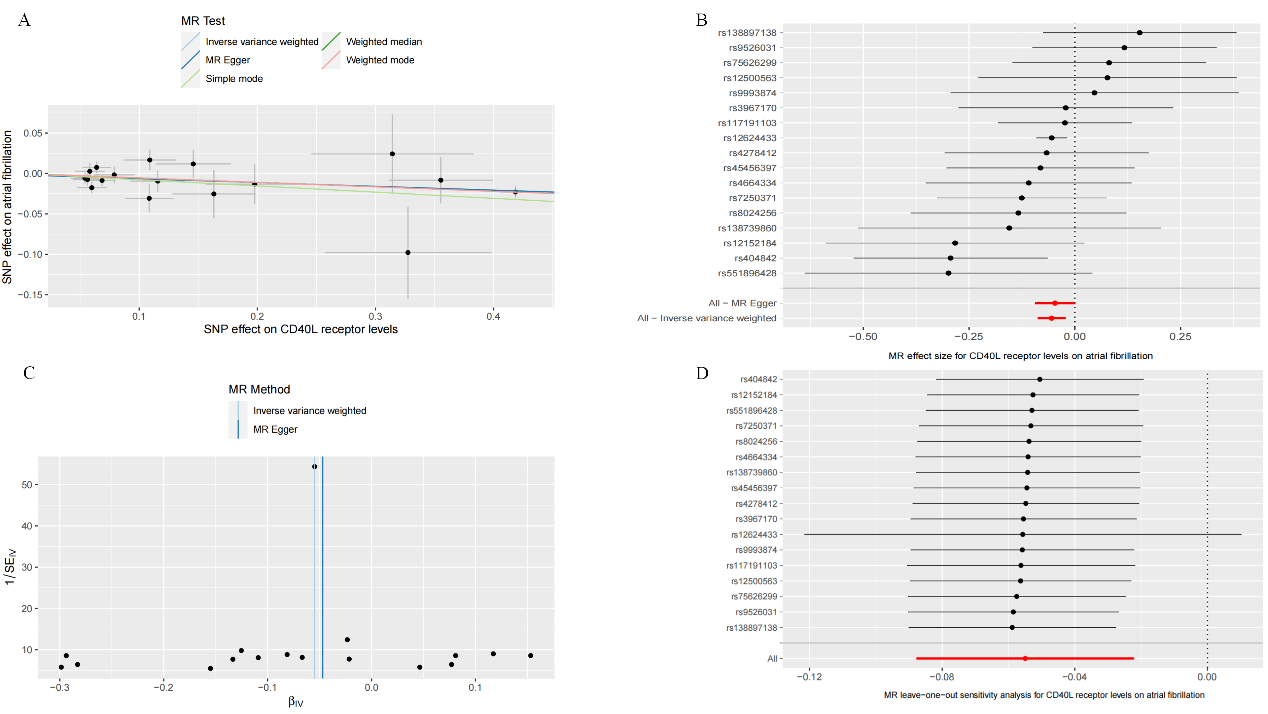
**

**Figure S3.** Mendelian randomization (MR) analysis of CD40L receptor and AF, when GWAS of AF from the study by Nielsen JB *et al.* were used as outcome. (A) Scatter plot of MR analysis; (B) Forest plot of MR analysis; (C) Funnel plots of MR analysis; (D) leave-one-out sensitivity analysis of MR analysis.

**Figure S4**

**
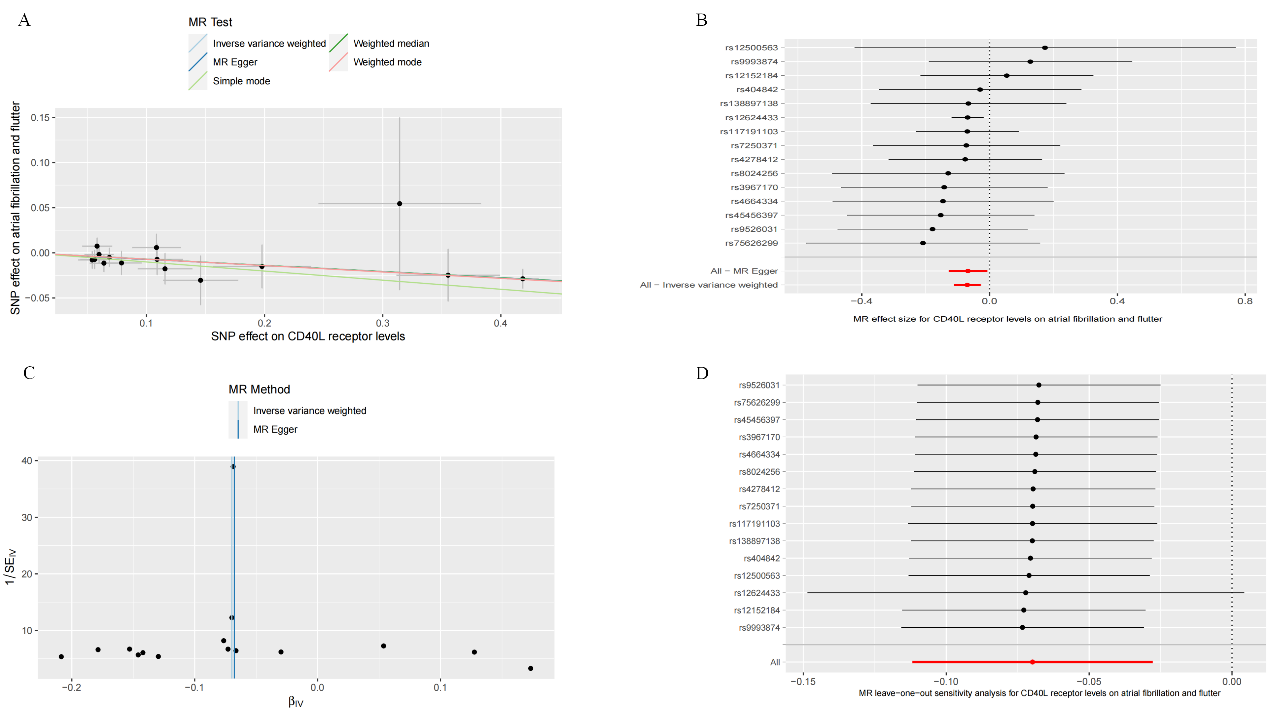
**

**Figure S4.** Mendelian randomization (MR) analysis of CD40L receptor and AF, when GWAS of AF from the FinnGen study were used as outcome. (A) Scatter plot of MR analysis; (B) Forest plot of MR analysis; (C) Funnel plots of MR analysis; (D) leave-one-out sensitivity analysis of MR analysis.

**Figure S5**

**
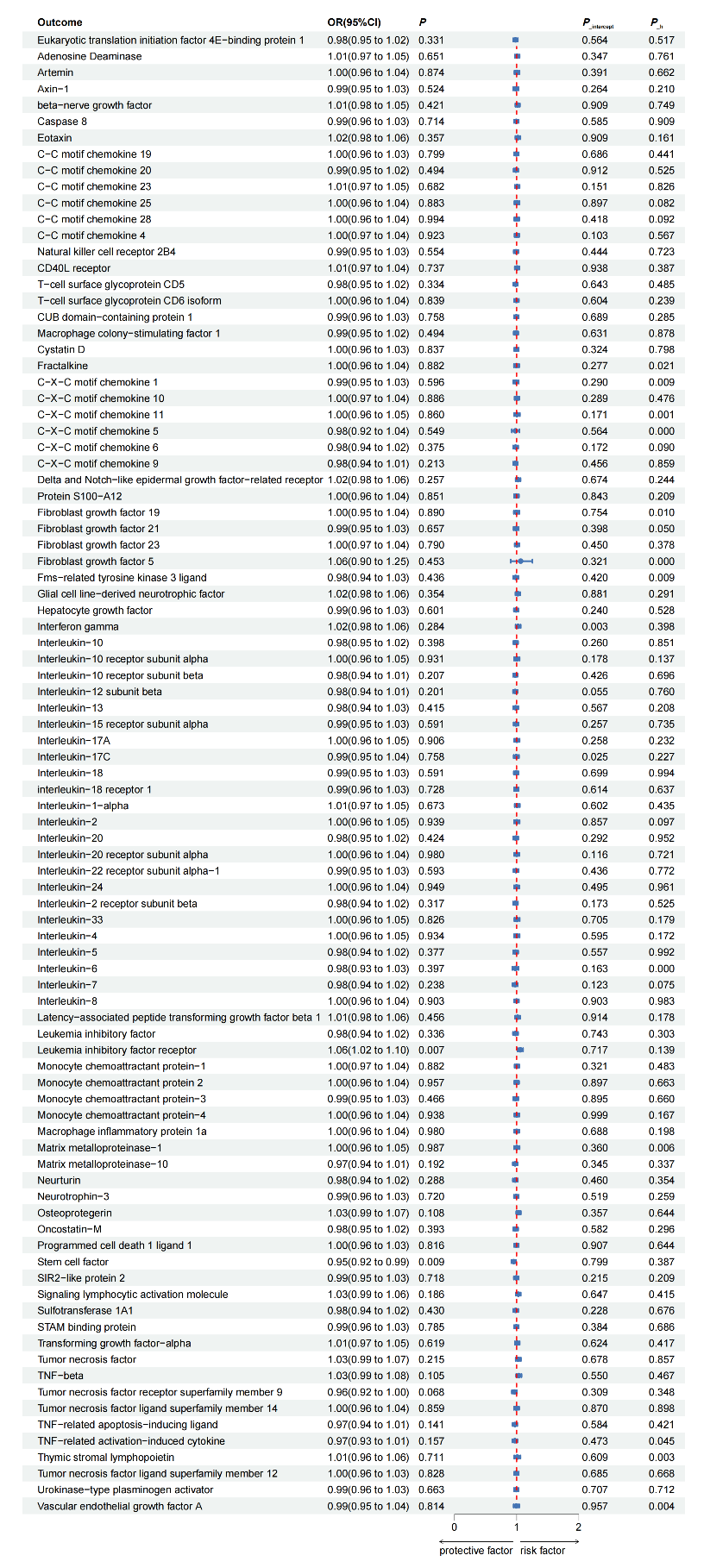
**

**Figure S5.** Causal correlations of atrial fibrillation on 91 inflammatory proteins, when GWAS of AF from the study by Nielsen JB *et al.* were used as exposure. AF, atrial fibrillation; CI, confidence interval; OR, odds ratio; SNP, single nucleotide polymorphism.

**Figure S6**

**
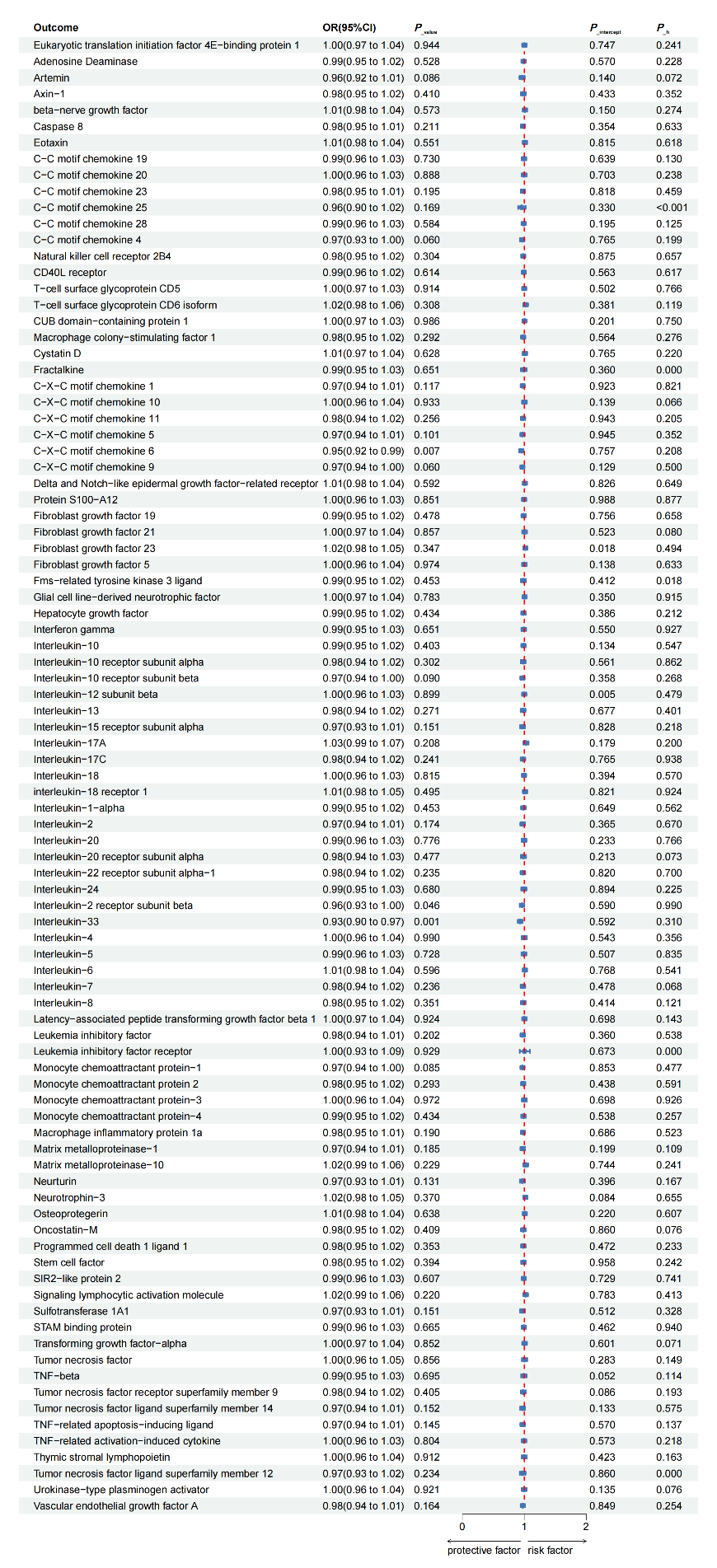
**

**Figure S6.** Secondary validation of the causal correlations of atrial fibrillation on 91 inflammatory proteins, when GWAS of AF from the FinnGen study were used as exposure. AF, atrial fibrillation; CI, confidence interval; OR, odds ratio; SNP, single nucleotide polymorphism.
